# Profiling, Distribution, and Risk Assessment of Parabens in Groundwater Near Dumpsites

**DOI:** 10.1021/acsomega.5c08260

**Published:** 2026-01-15

**Authors:** Esther A. Nnamani, Oluwaferanmi B. Otitoju, Ephraim Akor, Emmanuel I. Unuabonah, Martins O. Omorogie

**Affiliations:** † African Centre of Excellence for Water and Environmental Research (ACEWATER), Redeemer’s University, PMB 230, Ede 232101, Nigeria; ‡ Department of Chemical Sciences, Redeemer’s University, PMB 230, Ede 232101, Nigeria; § Chair of Urban Water Systems Engineering, Technical University of Munich, Am Coulombwall 3, D-85748 Garching, Germany

## Abstract

Over two billion people globally are predisposed to leachate infiltration of endocrine-disrupting compounds in groundwater sources, with parabens receiving great attention due to their widespread industrial applications. This study assessed the profiling, seasonal variability, and associated risks of five parabens: methyl (MeP), ethyl (EtP), propyl (PrP), butyl (BuP), and methyl-3,5-dichloro (McP) parabens in groundwater sources near dumpsites across rural and urban areas of Osun, Oyo, and Lagos States in Southwestern Nigeria. Samples were collected during the rainy and dry seasons. MeP and McP were the most prevalent parabens, with detection frequencies across the three states following this trend: MeP > McP > PrP > EtP > BuP. Seasonal average concentrations of parabens in GW were the highest in Osun State (45.52 mg/L rural/29.40 mg/L urban), followed by Oyo State (35.81 mg/L rural/22.26 mg/L urban), and Lagos State, which had the least (21.10 mg/L rural/28.24 mg/L urban). Concentrations were considerably higher during the dry season, possibly due to lower water tables and limited dilution. No notable difference was observed between rural and urban concentrations of paraben in groundwater samples, suggesting uniform exposure in both settings. Principal component analysis revealed similar anthropogenic influences, as indicated by the close clustering of paraben compounds across both seasons. Ecological risk assessments showed that invertebrates consistently exhibited acute and chronic ecological risk, with fish being the least affected. Human health assessments via Estimated Daily Intake, Chronic Daily Intake, and Hazard Quotient analyses demonstrated that children face significant health risks from paraben exposure, with Hazard index values significantly exceeding the safe threshold of (HI > 1) in most sites across both seasons. These findings are of alarming concern, especially with the high detection frequency of chlorinated paraben. Consequently, groundwater ecofriendly treatment and initiatives must be deployed, especially to underserved settings, as evident in this study.

## Graphical abstract

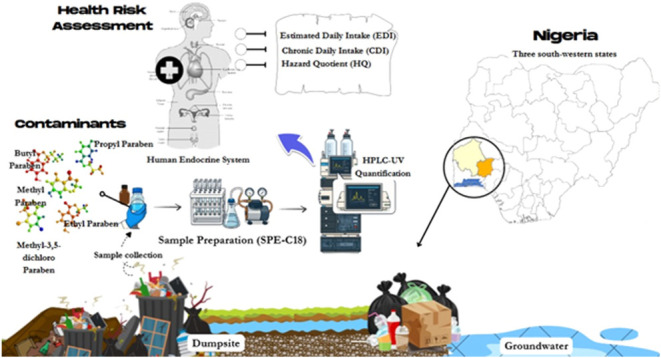

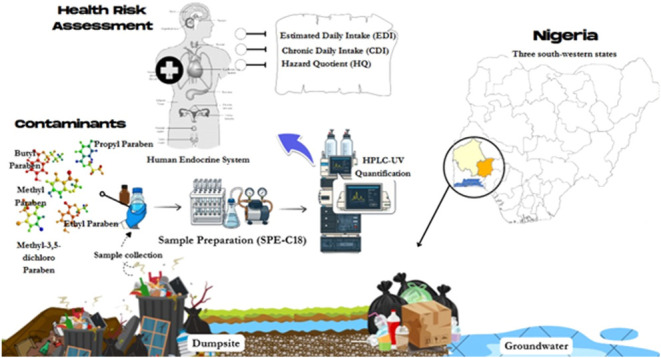

## 1 Introduction

Leachate infiltration from open dumpsites is gradually becoming a global environmental threat, posing significant risks to groundwater (GW) systems.^1^ Understanding the effect of open and unregulated dumpsites in GW is pivotal to safeguarding the quality of our drinking water supply.^2^ Leachate comprises of diverse organic and inorganic substances like heavy metals and endocrine-disrupting compounds (EDCs), such as personal care products, phenolics, and plasticizers.^3^ Urbanization and industrialization are largely responsible for increased open dumpsites, especially in developing nations.^4^ Urban and semiurban populations constitute 55% of the world population, and it is predicted to grow to 68% by 2050.^5^ In Africa, Nigeria is the most populated country, with very high industrial activities and minimal attention on the proper discharge of municipal and industrial waste.^6^ Consequently, contamination of water resources, especially drinking water supplies, is on the rise.^7^ Water sources, such as surface water (SW), are a convenient way to dispose of industrial waste; consequently, GW is often contaminated via leaching from dumping sites, contributing to waterborne diseases.^8^ Most of the dumpsites or landfills in developing nations are not engineered; therefore, they are constructed without detailed and well-layered liners, pipes, and containers.^9^ As a result, they lack monitoring facilities and have contributed significantly to leachate infiltration into GW systems.^10^ Dumpsite leachate is a combination of both organic and inorganic toxic compounds, formed from the interface between excess water percolating through layers of waste deposited in dumpsites.^11^ Since leachate from dumpsites is a direct source point for GW contamination by EDCs, it is noteworthy that GW sources close to dumpsites are prone to paraben contamination, a class of EDCs, because of their vast use as preservatives for both small- and large-scale production. Notable EDCs such as Polybrominated Diphenyl Ethers (PBDEs), heavy metals, polyfluorinated compounds, antibiotics, alkylphenols, and bisphenol A have been monitored and detected in and around open dumpsites at elevated concentrations, thereby posing a significant threat to GW quality.^2,11–14^ EDCs pollution in GW can be detected within a radius of 200–1000 m.^15^ They are known to be mutagenic, endocrine disruptors, and in some cases, carcinogenic.^16,17^

Parabens are a group of EDCs that are widely used as preservatives and antimicrobial substances in the food, pharmaceutical, and personal care industries.^18–21^ Elevated levels of these compounds have been reported in wastewater, SW, and GW, with high-frequency detections across various studies.^20,22,23^ The amount of parabens in food and cosmetic products can predispose humans to serious health concerns, such as obesity, reproductive and neurological disorders, via ingestion, dermal absorption, and inhalation in very extreme conditions.^24,25^ Prolonged exposure to parabens in the aquatic environment may lead to developmental disorders in aquatic organisms.^20,26^ Although dermal absorption of parabens has been reported as a potentially more critical route of human exposure due to its ability to bypass first-pass metabolism, ingestion typically results in extensive hydrolysis to 4-hydroxybenzoic acid, a more polar metabolite with reduced cell permeability, thereby leading to different toxicokinetic outcomes.^27,28^ Nevertheless, this study focuses on the ingestion of paraben-contaminated GW, which is a predominant source of drinking water in many communities. The most frequently used parabens, including methyl, ethyl, propyl, and butyl, are classified into two main groups based on the length of their alkyl chain. Short-chain alkyl length includes methyl and ethyl parabens, while long-chain alkyls encompass propyl and butyl parabens.^18,29^ During water disinfection processes involving chlorine, these compounds can undergo halogenation, leading to the formation of potentially toxic byproducts such as methyl-3,5-dichloro paraben and other chlorinated derivatives.^30,31^ Chlorinated parabens have been reported in wastewater and sludge, posing a serious challenge; however, there is a dearth of information on their presence in other environmental matrices, such as GW systems.^32^ These findings underscore the need for a more robust regulatory framework regarding the use of parabens globally. While China, Australia, Japan, and Europe have permissible limits for selected analogues of parabens, Africa is yet to set permissible limits, and this is largely attributed to the deficit of data in the region.^33^ The unregulated and often unconscious release of these EDCs when disposed of inappropriately can infiltrate the GW aquifer and make it unsafe for over 60% of the sub-Saharan region that relies on GW as its predominant drinking water supply.^34^

Considering the widespread use of parabens in industries and the unregulated disposal at both domestic and industrial levels in developing nations, it is imperative that comprehensive profiling be carried out to gain insight into the environmental fate of parabens in GW near dumpsites. Although, the targeted analytes in aquatic environments, including GW systems, have been investigated, there is a dearth of information on paraben pollution patterns near GW systems close to dumpsites globally. Additionally, parabens, owing to their structural stability and persistence, are likely to migrate through soil layers and contaminate GW near municipal landfills. This raises significant concern, as the consumption of paraben-contaminated GW poses serious health and ecological concerns.^23,33,35^

In this study, we aimed to investigate the geographical and seasonal variation in the profiling of parabens in GW sources close to dumpsites, as well as the corresponding health risks posed to both humans and aquatic organisms. The research focused on three Southwestern states in Nigeria, namely, Osun, Oyo, and Lagos, where GW sources serve as the principal source of drinking water. Targeted GW includes hand-dug wells, tap water, and boreholes around dumpsites within residential areas.^36^ The choice of parabens for the study is based on their widespread adoption as preservative and antimicrobial agents, especially in the personal care industry in Nigeria. Chlorination, an essential form of water treatment, necessitated the need for an investigative study on methyl-3,5-dichloro paraben as a potential halogenated byproduct.^31^ Physicochemical properties such as pH, total dissolved solids (TDS), and electric conductivity (EC) of the sampled GW were measured *in situ*, while principal component analysis was carried out on the measured physicochemical parameters and targeted analytes to ascertain the correlation between the studied analytes and the physicochemical parameters. Baseline data from this study will provide comprehensive information on the occurrence and distribution of parabens in GW near dumpsites. It will also support the development of a risk assessment framework to regulate paraben use, particularly by strengthening guidelines on allowable concentrations in consumer products and ensuring stricter oversight of their inclusion in over-the-counter formulations. To the best of our knowledge, there is currently limited regulatory oversight on the environmental and health impacts of halogenated paraben derivatives, underscoring the need for more stringent monitoring and policy interventions. This study will provide valuable insights for water treatment professionals in developing efficient remediation strategies for these persistent chemical contaminants in GW resources across Nigeria and Africa. Furthermore, it aligns with the United Nations Sustainable Development Goal 6, which emphasizes universal access to clean water and sanitation. Table S1 presents an overview of the targeted analytes examined in this study.

## 2 Materials and Methods

### 2.1 Standards and Reagents

Analytical standards of methyl paraben (methyl 4-hydroxybenzoate 99.7%), ethyl paraben (ethyl 4-hydroxybenzoate 99%), propyl paraben (propyl 4-hydroxybenzoate ≥99.0%), butyl paraben (butyl 4-hydroxybenzoate ≥99.0%), and methyl-3,5-dichloro paraben (methyl 3,5-dichlorobenzoate 99%) were purchased from Sigma-Aldrich (St. Louis, MO). HPLC-grade methanol and Oasis HLB SPE cartridges (500 mg, 6 mL) were purchased from Sigma-Aldrich (St. Louis, MO). Ultrapure water was obtained from Fisher Scientific. Standard stock solutions of each analyte (20 mg/L) were prepared in methanol and stored at 4 °C. Working solutions (1 mg/L) of mixed parabens were prepared by diluting the stock solution with ultrapure water before use.

### 2.2 Description of the Sampled Locations

GW samples were collected from the Osun, Oyo, and Lagos States, located in southwestern Nigeria, which lie within the tropical rainforest biome, characterized by high biodiversity and dense vegetation. Osun State spans approximately 14,875 km^2^ between latitudes 7°30′N and 4°30′E, while Oyo State covers about 28,454 km^2^ between latitudes 7°00′N and 9°00′N and longitudes 3°00′E and 5°00′E. Lagos State, though smaller at approximately 3577 km^2^, is densely populated and situated between latitudes 6°23′N and 6°41′N and longitudes 2°42′E and 3°42′E (Figure 1). Osun and Oyo States are known for their vast agricultural activities as well as a notable tie-dye industry. These states host a substantial number of small- and medium-sized enterprises (SMEs), engaging in various sectors including food processing, personal care, and pharmaceuticals. Lagos State serves as Nigeria’s commercial capital and hosts a vast array of industries ranging from manufacturing to services. The state accounts for over 53% of manufacturing employment in Nigeria, with industries including food and beverage, textiles, chemicals, and pharmaceutical and personal care materials. Open dumpsites in both rural and urban areas of these states, close to major residential and commercial activities, were identified. GW systems up to about 500 m from the already identified dumpsites were considered for this study. The coordinates of identified dumpsites and surrounding GW sources (Table S2) were recorded and utilized to develop the map shown in Figure 1.

**Figure 1:**
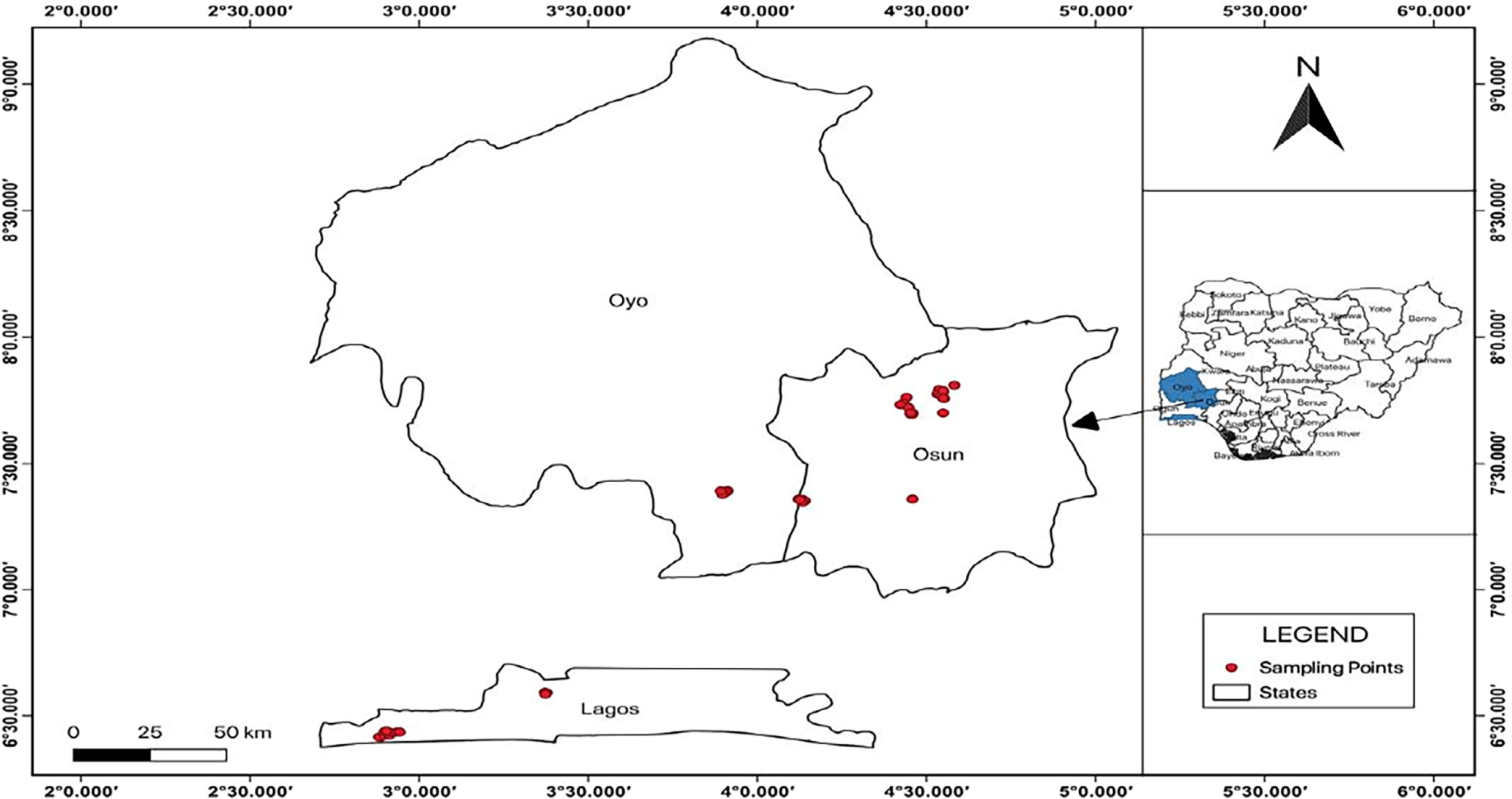
Map of sampling locations.

### 2.3 Groundwater Sampling Design

A one-year sampling campaign between May 2024 and April 2025 was carried out for the study from 60 different sites across the rural and urban areas of the three states, while considering the two main seasons in Nigeria, the dry and wet seasons. GW samples were collected from boreholes, taps, and hand-drill wells to a depth of about 18 m. Sample containers were precleaned in line with USEPA cleaning protocol and rinsed three times with the sample before introducing the final sample.^37,38^ Composite sampling (*n* = 3) was deployed for this study. For the hand-dug well, a clean container was lowered into the well to collect GW into sample containers and labeled appropriately. Samples from boreholes were introduced directly into the precleaned sample containers. Since the study aimed at evaluating the spatial variation of the targeted EDCs in GW, the sample design involved the collection of samples at intervals of 50–100 m within a 1 km radius from the dumpsites. Dumpsites around residential homes and in the proximity of GW sources were considered for the study. The TDS, pH, and EC of the collected samples were measured in situ using a calibrated HANNA pH/EC/TDS/Temperature (HI 9811-5) portable meter. Samples were stored in ice packs, transported to the laboratory, and preserved at 4 °C until further analysis. Field observation was done to understand prevalent anthropogenic activities and to substantiate the study’s outcome.

### 2.4 Sample Preparation and Extraction

Using a water filtration setup connected to a vacuum pump, water samples were filtered by using a 0.22 μm membrane filter. Afterward, filtered samples were pH-adjusted to pH 3.0. The solid phase extraction technique was employed to extract the targeted analytes from the sample matrix. Oasis HLB cartridges were preconditioned sequentially with 5 mL of HPLC-grade methanol and 5 mL of ultrapure water. Subsequently, 250 mL of each filtered sample spiked with a known concentration (1 mg/L) of the studied analytes (methyl, ethyl, propyl, butyl, and methylchloro parabens) was loaded onto the cartridges at a controlled flow rate of 8 mL/min. After the cartridges were washed with 5 mL of ultrapure water and vacuum-dried for 15 min, analytes were eluted with 5 mL of HPLC-grade methanol at a regulated flow rate of 1 mL/min. The eluate was then evaporated to near dryness at 50 °C, reconstituted in 0.5 mL of HPLC-grade methanol, and filtered through a 0.22 μm organic-phase needle filter. Processed samples were stored at 4 °C until instrumental analysis.

### 2.5 Instrumental Analysis

An optimized method was developed for both quantitative and qualitative analysis using a high-performance liquid chromatography-ultraviolet detector (Agilent Series 1100 LC system, Agilent Technologies, Germany). Peak separation was done using C-18 (25 cm × 4.6 mm, 5 μm particle size). Sample injection was done via an autosampler at a flow rate of 0.7 mL/min with column temperature of 30 °C. The mobile phase consisted of HPLC-grade methanol and water in a 70:30 (v/v) ratio, operated under isocratic elution conditions. The injection volume was set to 20 μL with a total run time of 18 min. The four targeted analytes (methyl, ethyl, propyl, and butyl parabens) were detected at 254 nm, whereas methylchloro paraben was detected at 280 nm.^22,39^

### 2.6 Quality Control and Quality Assurance

In order to ensure data reliability and analytical precision, rigorous quality control and assurance measures were implemented throughout the sampling and analysis process. All glassware and equipment used for this study were thoroughly cleaned to avoid possible contamination or a false representation of the studied sites. Before each sample injection, the autosampler was rinsed with HPLC-grade methanol to eliminate the sample carryover. Blank samples (field, solvent, laboratory, and equipment) were analyzed procedurally to prevent matrix interference for each batch of 10 samples. Results obtained from this study were blank-deducted. Midpoint calibration and methanol blank were introduced to monitor any drift in the instrument’s response and probable cross-contamination. Individual standard solution of each compound (20 mg/L) was introduced into the equipment to identify the optimal wavelength for each analyte. Quantification of analytes was performed using individual paraben standards, with standard calibration curves obtained by analyzing aqueous solutions containing the analytes of interest, ranging from a concentration of 0.01 to 20 mg/L, with acceptable coefficient (*r* ^2^) values as shown in Table 1. Instrument’s limit of detection (LOD) and quantification (LOQ) were estimated as three times the signal-to-noise ratio using the standard deviation of the 7-point calibration intercept divided by the slope, while LOQ is 10 times the ratio. To evaluate the accuracy of the method, absolute recovery was carried out by spiking the samples with mixed paraben standards before the SPE procedure at low (2 mg/L), mid (12.5 mg/L), and high (25 mg/L). Samples were prepared in triplicate, and absolute recovery was computed as the ratio of measured concentration to known spiked concentration and expressed as a percentage. The method’s precision was evaluated in terms of relative standard deviation (RSD) to assess the reproducibility and repeatability of the results, and all were below the permissible limit, hence validating the optimized method. To maintain instrument performance and data quality, system suitability parameters, including retention time consistency, peak shape, and resolution, were monitored throughout the analysis. Additionally, samples were stored in amber vials at 4 °C and analyzed within 48 h to minimize the analyte degradation.

**Table 1:** Instrumental and Analytical Validation for Paraben Analysis in GW Using a High-Performance Liquid Chromatography-Ultraviolet (HPLC-UV) Detector

paraben	retention time (min)	*R* ^2^	linear range (mg/L)	LOD (mg/L)	LOQ (mg/L)	spiked conc. (mg/L)	% recovery ± SD	precision (RSD)
MeP	7.8	0.9992	0.1–20	0.46	1.54	2	99.3 ± 1.6	2.9
						12.5	95.3 ± 3.4	6.3
						25	97.9 ± 1.9	3.5
EtP	8.9	0.9992	0.1–20	0.09	0.32	2	98.6 ± 4.43	7.9
						12.5	97.4 ± 2.3	4.1
						25	97.3 ± 1.8	3.2
PrP	10.9	0.9993	0.1–20	0.079	0.263	2	99.1 ± 1.4	2.6
						12.5	102 ± 3.7	6.3
						25	97.5 ± 3.3	5.8
BuP	14.3	0.9971	0.1–20	0.26	0.87	2	94.9 ± 3.2	5.9
						12.5	101.9 ± 0.9	1.6
						25	96.8 ± 2.1	3.8
McP	5.6	0.9974	0.1–20	0.09	0.29	2	99.8 ± 2.7	4.9
						12.5	98.2 ± 3.5	6.1
						25	99.3 ± 1.9	3.4

### 2.7 Environmental Risk Assessment

#### 2.7.1 Ecological Risk Assessment

Parabens have been scientifically validated to cause reproductive and developmental disorders in aquatic organisms.^22,33,40,41^ Given their persistence in the environment and potential bioaccumulation, it is crucial to compute the predicted risk of exposure of aquatic organisms to these recalcitrant compounds. In this study, the risk quotient (RQ) for three trophic levels, namely algae, invertebrates (daphnia), and fish, was considered. The RQ was estimated based on the parabens measured concentration in the collected samples using this equation:

1 $$\mathrm{RQ}=\frac{\mathrm{MEC}}{\mathrm{PNEC}}$$

Where MEC is the measured environmental concentration, and PNEC is the predicted no-effect concentration. The PNEC value was calculated for both acute and chronic tests using their respective median effect/lethal concentration (EC_50_/LC_50_) and no-observed effect concentration (NOEC) divided by an assessment factor (AF). Toxicity data for this study were obtained from the 2011 USEPA Ecotoxicology database, literature, and Tier II environmental assessment for parabens.^42^ RQ values obtained from this were classified into three levels: RQ > 1, indicating a high ecological risk; RQ values between 0.1 < RQ < 1 signifying a median risk, and RQ < 0.1 suggesting a minimal ecological risk.Table S3 shows the values used in computing ecological risk to the three key trophic levels.

2 $${\mathrm{PNE}C}_{\mathrm{acute}}=\frac{{\mathrm{LC}}_{50}\;\mathrm{or}\;{\mathrm{EC}}_{50}}{\mathrm{AF}};,\mathrm{AF value}=100$$

3 $${\mathrm{PNEC}}_{\mathrm{chronic}}=\frac{\mathrm{NOECi}}{\mathrm{AF}};,\mathrm{AF value}=10$$

#### 2.7.2 Human Risk Assessment

Potential human health impacts of parabens detected in GW in this study were evaluated by computing the estimated daily intake (EDI), chronic daily intake (CDI), and hazard quotient (HQ) according to the equations below. EDI gave insight into the risks exposed by an individual (children or adults) daily. CDI, which signifies the lifetime average daily dose of exposure to a contaminant, was used to estimate both cancer and noncarcinogenic risk for children and adults, as no cancer slope factor has been established for parabens by the United States for Environmental Protection Agency.^43^ HQ was used to assess human health exposure to parabens via a single exposure pathway. As a result of multiple co-occurrences of these paraben compounds in the collected sample, the Hazard Index (HI), which estimates the probable harm caused by multiple exposures via individual HQ, was computed in this study. Parameters for evaluating human health risk are detailed in Table 2.

4 $$\mathrm{EDI}\;\left(\mathrm{mg}/\mathrm{kg}/\mathrm{day}\right)=\frac{\left(C\times \mathrm{IR}\right)}{\mathrm{BW}}$$

$$CDI\,=\frac{\left(C\times DR\times EF\times ED\right)}{\left(BW\times AT\right)}$$

6 $$\mathrm{HQ}=\frac{\mathrm{EDI}}{\mathrm{RfD}}$$

7 HI=sum of hazard
quotients

**Table 2:** Parameters for the Evaluation of Human Health Risk Assessment of Paraben in GW Near Dumpsites

parameters	symbol	unit	value (adult/children)	ref
concentration	C	mg/L		
water ingestion rate	IR	L/day	2/1	^44^
body weight	BW	kg	70/15	^11,45^
exposure frequency	EF	day/year	365/365	^46^
exposure duration	ED	years	30/6	^46^
average exposure time	AT	days	10.950/2190	^45,46^
oral reference dose for MeP	RfD	mg/kg/day	10	^42^
oral reference dose for EtP	RfD	mg/kg/day	10	^42^
oral reference dose for PrP	RfD	mg/kg/day	0.1	^42^
oral reference dose for BuP	RfD	mg/kg/day	0.16	^47^
oral reference dose for McP	RfD	mg/kg/day		

### 2.8 Data Analysis and Evaluation

Physiochemical characterization, detection frequency, and seasonal variation in paraben contamination across the three states were analyzed using GraphPad Prism (2024). Statistical comparisons of parabens concentration among the Osun, Oyo, and Lagos States were performed with the nonparametric Mann–Whitney *U* test in IBM Statistical Package for the Social Sciences (SPSS) Statistics v21. Additionally, the Kruskal–Wallis test was employed to compare the concentrations of parabens between rural and urban sampling sites. Nonparametric Spearman correlation analysis was conducted to examine the correlation among individual paraben concentrations. Statistical significance was set at *p* < 0.05. Multivariate statistical analysis was assessed using the principal component analysis (PCA) software to evaluate relationships among the targeted analytes.^22^ PCA is a commonly applied tool for multivariate statistics in environmental analysis and has been used by several researchers to detect the correlation between different ecological variables and the quality of the data set.^36,48^ It is also an important tool for source apportionment of the targeted analytes.^49,50^ Hierarchical cluster analysis (HCA) was performed on standardized physiochemical parameters and paraben concentrations in GW samples to validate observed patterns in PCA. The variables were normalized to zero mean and unit variance to ensure no disparity in weighting. Euclidean distance was utilized as the similarity index, and clusters were formed using the average linkage method.

## 3 Results and Discussion

### 3.1 Physicochemical Characterization of GW Near Dumpsites

Essential physicochemical parameters assessed in GW samples during the dry and rain seasons included pH, electrical conductivity (EC), and total dissolved solids (TDS). Results for EC and TDS are presented in Figure 2, while pH data are represented in Figure S1. These data sets provide spatial (rural vs urban) and seasonal (rainy vs dry) variations across the three states.

**Figure 2:**
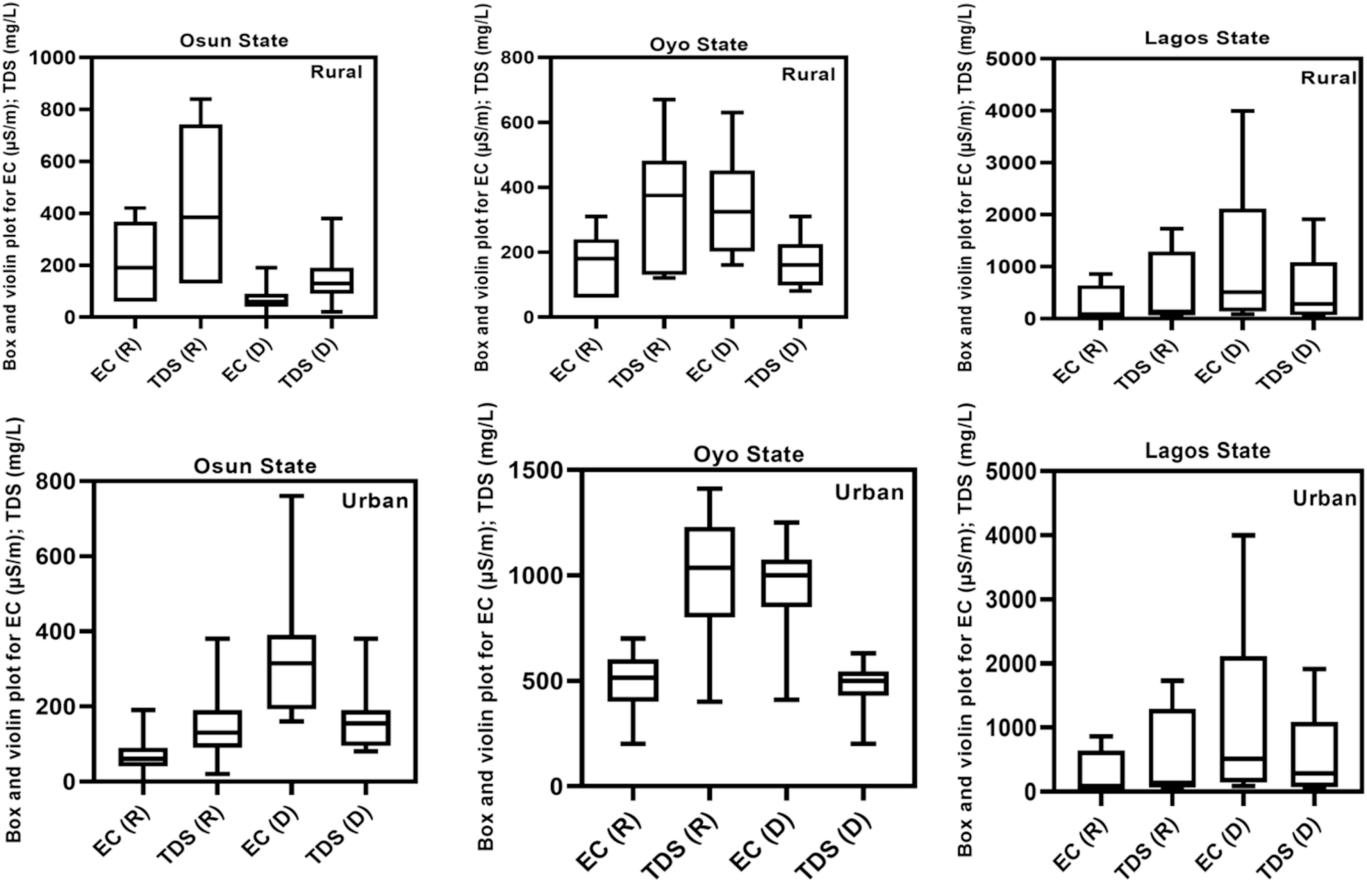
Box and Violin Plot for EC and TDS in GW samples from the Osun, Oyo, and Lagos States for both rainy and dry seasons in rural and urban areas (R= Rainy season; D= Dry season; EC= Electric conductivity; TDS= Total Dissolved Solids).

The values of the EC obtained for the majority of the sampled locations were within the permissible standards of 500 μS/cm prescribed by the World Health Organization (WHO).^36^ In rural areas, mean EC values for the rainy and dry seasons were 437 and 569 μS/cm (Osun), 341 and 350 μS/cm (Oyo), and 119 and 139 μS/cm (Lagos), respectively. In urban areas, corresponding EC values were 273 and 336 μS/cm (Osun), 471 and 949 μS/cm (Oyo), and 591 and 1,096 μS/cm (Lagos). Exceedances of the WHO limit occurred in Osun (rural, dry season), Oyo (urban, dry season), and Lagos (urban, rainy, and dry seasons). EC values were consistently higher during the dry season, which was attributable to limited rainfall, evapoconcentration, and consequent accumulation of dissolved ions.

The value of the TDS followed similar spatial and seasonal patterns. In rural areas, mean TDS values for the rainy and dry seasons were 215 and 283 mg/L (Osun), 164 and 173 mg/L (Oyo), and 56 and 140 mg/L (Lagos). Urban TDS concentrations ranged from 155 and 185 mg/L (Osun), from 232 and 475 mg/L (Oyo), to 289 and 511 mg/L (Lagos) in the rainy and dry seasons, respectively. All measured values were below the WHO permissible limit of 1000 mg/L.^51^ However, some values, especially in urban areas during the dry season, approached and slightly exceeded the Nigerian Standard for Drinking Water Quality (NSDWQ).^52,53^ As previously reported, TDS levels were higher during the dry season across most locations. Urban areas, particularly in Lagos and Oyo States, had higher TDS values than rural areas, and this disparity may be due to increased anthropogenic activities, such as industrial discharge and urban surface runoff.

The pH values of the GW samples fell within the WHO recommended range of 6.5–8.5 for potable water.^53,54^ Mean rural pH values during the rainy and dry seasons were 7.7 and 6.7 (Osun), 8.2 and 6.9 (Oyo), and 7.1 and 7.4 (Lagos). In urban areas, the corresponding pH values were 7.7 and 6.7 (Osun), 8.5 and 6.9 (Oyo), and 7.9 and 6.8 (Lagos). The pH was consistently higher during the rainy season, reflecting increased dissolution of atmospheric gases and organic matter, which enhances buffering capacity and slightly elevates pH. Borehole samples generally exhibited lower pH values than well water, consistent with literature reports that deeper aquifers possess weaker buffering capacity due to reduced interaction with carbonate-bearing surface materials.^36,55^

The target analytes for this study are weak acids with p*K*_a_ values between 8.17 and 9.96, suggesting that their extraction efficiency is pH-dependent. Consequently, pH adjustment was performed to enhance extraction efficiency. In this study, GW near dumpsites showed three consistent trends: seasonal dilution–concentration cycles affecting ionic strength and buffering, higher mineralization in urban compared to rural sites from anthropogenic loading, and geochemical conditions influencing the persistence and extractability of parabens.^56,57^ These patterns underscore the importance of monitoring baseline hydrochemical indicators when assessing EDCs in GW.

### 3.2 Detection and Distribution Frequency of Paraben Compounds in GW

The concentrations reported in this study were substantially higher than what has been reported in other studies.^58^ Aside from the proximity of the sampled GW to open dumpsites, several factors likely contribute to the observed high paraben concentrations. Insights from oral interviews and field observations indicate that all of the open dump sites are poorly constructed and lack proper engineering controls, which facilitate leachate infiltration into surrounding GW. The extensive use of parabens as antimicrobial preservatives in the region, coupled with improper disposal practices by unregulated small-scale businesses, further amplifies the input of these compounds into the environment. Moreover, the age of the dumpsites may enhance leachate generation and promote the gradual release of accumulated contaminant affirming elevated concentration in nearby GW.^59^

There was 100% detection frequency for some paraben compounds across the three states, as depicted in Figure S2. Detection frequencies were generally higher in urban areas during the dry season compared to those in rural areas across both seasons, suggesting that higher population density, greater usage of paraben-containing products, and limited rainfall-driven dilution may exacerbate groundwater contamination. Ethyl paraben (EtP) and butyl paraben (BuP) had 0% detection frequency in rural settings, while BuP only had 0% detection frequency in urban areas. Among the rural areas, Osun recorded the highest detection frequencies, likely influenced by its relatively lower socioeconomic lifestyles and unregulated access to these antimicrobial preservatives compared to other studied rural areas that are more enlightened. In the Urban areas, Lagos recorded the highest detection rates overall, which is expected given its location as the largest concentration of chemical shops in West Africa. The elevated detection during dry seasons may reflect diminished dilution capacity, while the disparate rural–urban patterns underscore the combined influence of socioeconomic factors and regulatory environments.

The mean concentrations of parabens across seasons in GW samples near dumpsites are presented in Figure 3, while Figure 4 shows box plots illustrating the mean, median, maximum, and minimum values for seasonal distributions in rural and urban areas of the states of Osun, Oyo, and Lagos States, Nigeria. From Figure 3, across the three states, the mean concentrations of parabens were higher in the dry season than in the rainy season. This pattern is consistent with seasonal recharge dynamics, where reduced recharge during the dry season limits dilution and flushing, culminating to increased contaminant concentration in GW.^60,61^

**Figure 3:**
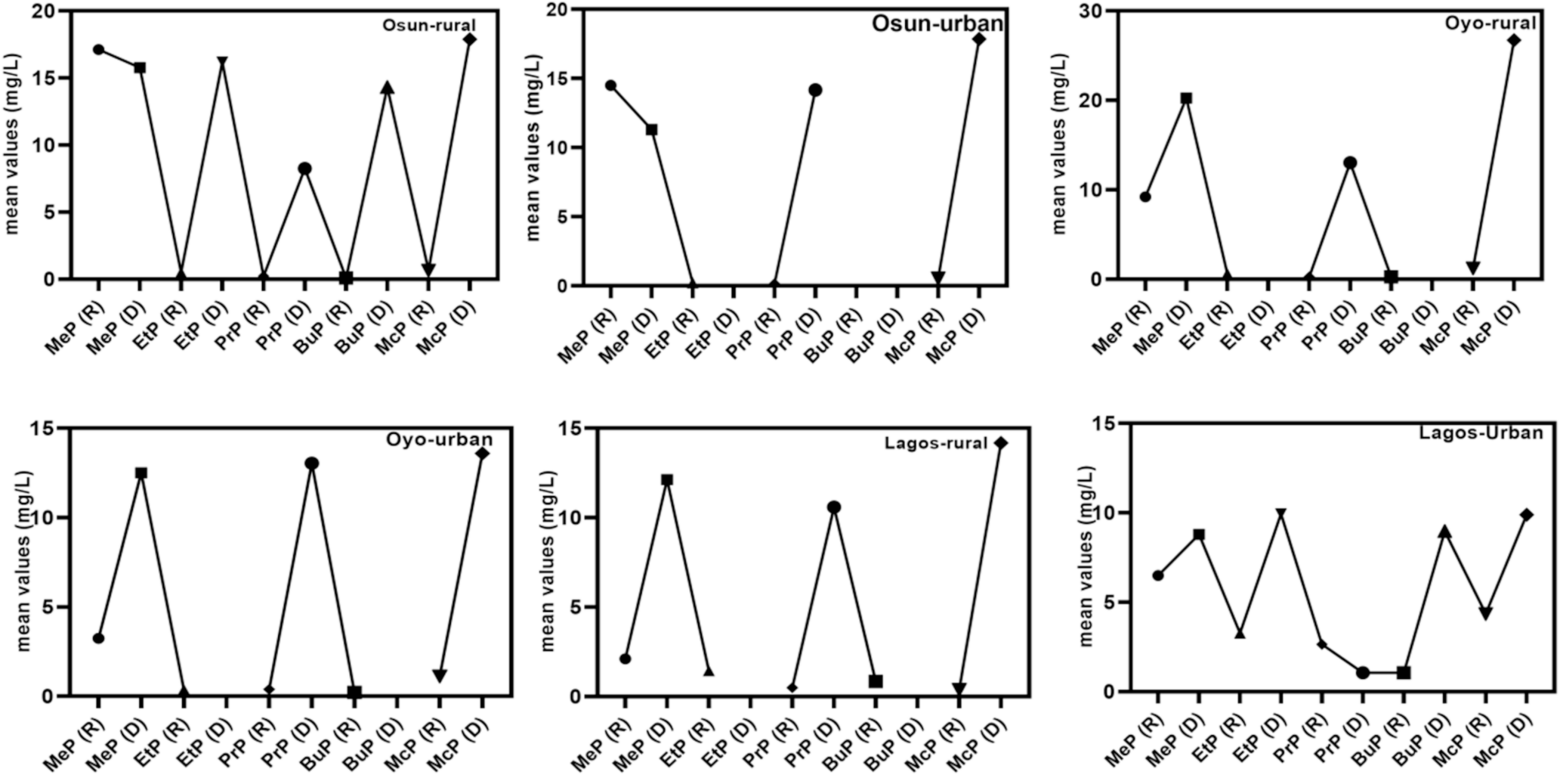
Mean Seasonal Concentrations of Parabens in the Osun, Oyo, and Lagos GW Samples in Nigeria.

**Figure 4:**
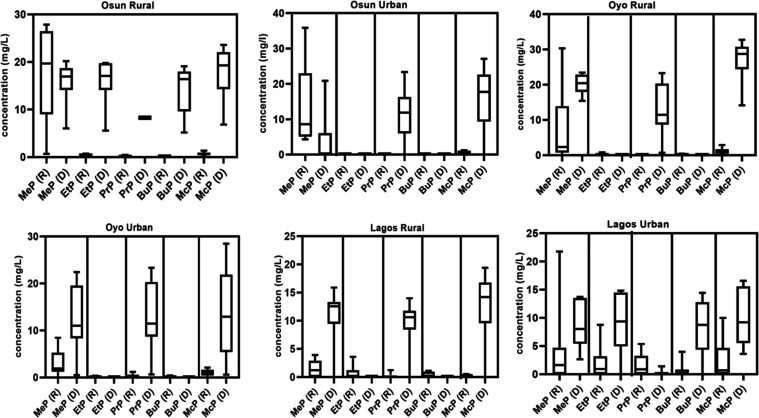
Box and whisker plot for Paraben Distribution in rural and urban areas of the Osun, Oyo, and Lagos States of Nigeria for both rainy (R) and dry (D) seasons (MeP = methylparaben; EtP= Ethylparaben; PrP= Propylparaben; BuP= butylparaben; McP= Methyl-3,5-dichloroparaben).

According to the study areas, rural GW sources generally contained higher concentrations of parabens than their urban counterparts, particularly for the Osun and Oyo States, indicative of greater vulnerability of rural aquifers due to uncontrolled waste disposal and shallow well depths that favor infiltration. In contrast, Lagos displayed an inverse pattern with higher concentrations in urban locations, reflecting the intense anthropogenic pressures and industrial discharges characteristic of its urban environment.

Methyl paraben (MeP) and methyl-3-5-dichloro paraben (McP) dominated the paraben profile in all samples, showing concentrations approximately 2-fold higher than those of ethyl paraben (EtP), propyl paraben (PrP), and butyl paraben (BuP), which were present at comparatively lower and similar levels. This distribution indicates a clear prevalence of short-chain parabens, particularly MeP and McP, in the samples (McP ≈ MeP ≫ PrP ≈ EtP ≈ BuP). Their codominance is attributed to their high solubility and frequent use in personal care and food products, as well as McP’s enhanced persistence due to chlorination in domestic water systems.^62,63^ MeP consistently recorded the highest mean concentrations across both seasons, with peak values in the dry season (20.27 mg/L in Oyo Rural and 15.78 mg/L in Osun Rural) compared to 9.20 and 17.13 mg/L in the rainy season, as shown in Figures 3 and 4. Its dominance reflects its high mobility, hydrophilic nature, and widespread use in food, pharmaceuticals, and personal care products.^64,65^

Methyl-3-5-dichloro paraben (McP) was the second most frequently detected paraben in this study. Its persistence is likely due to its reaction with free chlorine in water systems to form halogenated byproducts, which are more stable, persistent, and potentially toxic than their parent compounds.^31,66^ The reliance on chlorination for water disinfection in the study areas may therefore enhance McP’s persistence in GW. Although PrP concentrations were lower than those of MeP and McP, they represent the highest reported in Nigeria to date.^22,23,65^ and even exceeded values documented in Egypt.^14^ EtP showed a sharp increase in the dry season, particularly in rural samples, while BuP concentrations were mostly below detection limits, consistent with its low water solubility and higher octanol–water partition coefficient (log *K*_OW_), which favors sorption onto soil particles.

Ethyl paraben (EtP) and propyl paraben (PrP) exhibited comparable detection frequencies in the Osun and Lagos States, whereas EtP was slightly more in the Oyo. Across both seasons and environments, all parabens were detected except BuP, which was below detection limits during the dry season. In Lagos, EtP, PrP, and BuP shared identical detection frequencies, while in Oyo, PrP and BuP occurred at the same frequency. These variations reflect the combined effects of seasonal dynamics and urban–rural differences on the occurrence and distribution of parabens in GW systems.The Kruskal–Wallis test revealed no statistically significant difference (*p* > 0.05) in the average concentration of these paraben compounds across the three states in both rural and urban settings. This is suggestive of the fact that people in both settings are predisposed to these endocrine-disrupting compounds in the surrounding GW sources.

Spatial and seasonal variations also shape ecological risks. GW from Oyo and Lagos posed higher risks to algae, invertebrates, and fish during the dry season due to elevated concentrations and reduced aquifer dilution, whereas GW from Osun presented greater risks in the rainy season, likely due to shallow hand-dug wells susceptible to surface runoff. Rural GW generally had higher mean total concentrations than urban samples (45.52 mg/L vs 29.40 mg/L in Osun; 35.81 mg/L vs 22.26 mg/L in Oyo), although Lagos showed the reverse pattern (28.24 mg/L urban; 21.10 mg/L rural). These findings indicate greater contamination risks in rural communities arising from poor waste management, direct waste discharge, and inadequate treatment.^67,68^ The elevated concentrations observed, which exceeded those previously reported in Nigeria and other countries (Table 3), likely stem from dumpsite proximity, improper waste management practices, and leachate infiltration. Overall, the data spread was widest in Lagos, moderate in Osun, and relatively narrow in Oyo, reflecting differing environmental factors and land-use influences on GW quality across the states. Furthermore, the results demonstrate how seasonal recharge dynamics, land-use practices, and aquifer geochemistry collectively regulate the distribution and persistence of parabens in GW systems.

**Table 3:** Comparative Concentration of Parabens (mg/L) in GW from This Present Study and Previous Reports^a^

country	GW system	MeP	EtP	PrP	BuP	McP	ref
Nigeria	wells, borehole	ND–20.27	ND–16.19	ND–13.04	ND–14.31	0.57–26.73	this study
Nigeria	wells, borehole	ND–0.212	ND–0.210	ND–0.217	ND–0.293	NS	^22^
Nigeria	wells	ND–0.342	ND–0.295	ND–0.299	ND–0.400	NS	^39^
Nigeria	well, boreholes	0.054–0.153	0.078–0.148	0.0213–0.0832	0.0755–0.0801	NS	^65^
India	well	ND	ND	ND–0.0123	ND–0.005	NS	^69^
China	wells	0.006/0.083	0.0016/0.0125	0.0009/0.0225	ND	NS	^70^
Egypt	wells	MDL–0.0472		MDL–0.050	MDL–0.0258	NS	^14^
Poland	wells	MDL–0.0543	NS	NS		NS	^64^

a ND (not detected); (not monitored/reported); MDL (minimum detection limit); NS (not studied).

### 3.3 Multivariate Statistics

#### 3.3.1 Principal Component Analysis

The results of the principal component analysis (PCA) for the studied parameters across both rural and urban settings in the Osun, Oyo, and Lagos States are presented in Table S4. Data suitability was confirmed by the Kaiser–Meyer–Olkin (KMO) test (≥0.5) and Bartlett’s Test of Sphericity (*p* < 0.001), validating PCA application. Across states, three to four principal components explained between 66% and 86% of the total variance, with EC and TDS consistently showing high positive loadings (≥0.80–0.97). This strong association is expected, given their close physicochemical relationship, which has been widely reported.^46,71–73^ Also, their consistent coloading across sites suggests that ionic strength is a significant hydrogeochemical factor that influence GW variability. For the Osun GW, three PCs explained 86.02% of the variance in rural and 66.00% in urban samples. In both areas, PC1 was dominated by EC and TDS (≥0.82), confirming that ionic strength strongly influences GW. In rural areas, ethyl paraben (EtP) and butyl paraben (BuP) during dry season loaded positively with EtP_D (0.79) and BuP_D (0.81) on PC1, whereas their rainy season counterparts EtP_R (−0.65) and BuP_R (−0.95) loaded negatively, indicating seasonal differences in the occurrence of parabens. This contrasting behavior of BuP across seasons confirms that seasonal shift PC1 showcases seasonal variability, controlled by ionic strength. PC2 showed strong negative loadings for methyl paraben (MeP) and propyl paraben (PrP) during the rainy season (MeP_R = −0.94, and PrP_R = −0.91), revealing distinct rainy-season patterns for these compounds. PC3 reflected a moderate positive loading for methyl-3-5-dichloro paraben (McP_R = 0.70 and MeP_D = 0.68), reflecting a secondary variability trend shared by these compounds across the seasons. However, this observed pattern does not indicate possibly transformation but a covarying behavior driven by similar anthropogenic activities. In the urban region of the Osun, variance was lower and there were no strong or consistent paraben groupings across the PCs. This suggests an intense contamination pattern influence by site-specific conditions.

For the Oyo rural GW four PCs explained 78.53% cumulative variance. PC1 was dominated by EC (0.87), TDS (0.88), PrP_R (0.69), BuP_R (0.67), and BuP_D (0.73), reflecting a broad association between water chemistry and the longer-chain parabens, plausibly linked to their moderate hydrophobicity and slower degradation rates. Shorter-chain parabens such as MeP_R (−0.45), MeP_D (−0.31), and PrP_D (−0.48) loaded negatively, suggesting either faster environmental degradation or different pollution source points. PC2 was dominated by McP_R (0.87), highlighting the rainy-season prevalence of this compound. PC3 exhibited strong associations of EtP_D (0.89) and PrP_D (0.89), indicative of accumulation during the dry season, whereas PC4 showed strong positive loading between EtP_R (0.89) and McP_D (0.80), which may reflect the persistence of chlorinated derivatives, consistent with their increased lipophilicity. 80.64% of the cumulative variance was explained in Oyo urban GW. PCA patterns were more dispersed, reflecting heterogeneous inputs. PC1 (26.91%) grouped MeP_R (0.89), EtP_R (0.95), PrP_R (0.53), and MeP_D (0.95), consistent with widespread use of personal care products and potential leachate contributions. PC2 was dominated by EC (0.96), TDS (0.97), EtP_D (0.96), and BuP_D (0.77), supporting a generalized relationship between parabens, particularly for EtP, BuP and ionic strength in GW. PC3 (BuP_R 0.91; McP_R 0.90) and PC4 (McP_D 0.90; EtP_D 0.63) suggest covarying behavior of compounds with similar physicochemical properties rather than direct transformation processes. The repeated coloading of EtP and BuP with EC and TDS across sites highlights a broader trend of parabens being tied to water chemistry, while variations in shorter-chain parabens reflect differential solubility, hydrophobicity, and seasonality.

In Lagos rural GW, three PCs explained 81.55% of the variance. PC1 (42.62%) depicts positive correlation for EtP_D (0.90), BuP_D (0.87), and PrP_R (0.81) but negative correlation for EtP_R (−0.83) and MeP_D (−0.86). This points to opposite seasonal behaviors in paraben accumulation. PC2 (29.08%) was dominated by BuP_R (0.89), pH (0.90), and TDS (0.89), suggesting geochemical influence, while PC3 (9.86%) highlighted PrP_D (0.90), reflecting compound-specific variation. For Lagos urban GW, three PCs explained 86.00% of the variance. PC1 (46.76%) had strong loadings for MeP_R (0.93), EtP_R (0.97), PrP_R (0.94), EtP_D (0.96), BuP_D (0.84), pH (0.74), and EC (0.72), pointing to widespread paraben presence influence by the sampled water chemistry. PC2 (23.56%) grouped EtP_R (0.97), BuP_R (0.81), PrP_D (0.81), and McP_R (0.86), while PC3 (15.71%) showed positive loadings for PrP_R (0.94) and MeP_R (0.93) with negative hydrochemical parameters, indicating the compound stability under lower ionic conditions. Despite cumulative variances exceeding the ≥ 65% threshold, PCA did not reveal discrete, uniform clusters. Instead, the results highlight complex, overlapping, and site-specific associations.

The 3D plots in Figure 5A–F visually complement the statistical PCA results. In the model of Osun (Figure 5A,B), EC, TDS, and pH cluster closely across rural and urban samples, confirming their influence, while MeP_R and PrP_R are positioned farther away, reflecting distinct contamination pathways. In the case of Oyo (Figure 5C,D), longer-chain parabens (BuP_R, BuP_D, PrP_R) cluster with EC and TDS in rural sites, whereas MeP_R and EtP_R are separated, consistent with their negative loadings in PC1. Urban Oyo shows a similar clustering pattern of BuP_D and McP_R near EC/TDS, highlighting shared hydrophobicity and solute-driven behavior. In Lagos (Figure 5E,F), EC and TDS dominate both rural and urban samples, while paraben positions vary by compound and season. BuP_R and EtP_D cluster with EC/TDS in rural samples, whereas MeP_R and PrP_R are more dispersed. In urban Lagos, McP_R and BuP_D cluster near EC/TDS, reflecting hydrophobic and persistent compounds under a higher ionic strength. The 3D plots corroborate that GW variability is primarily influenced by EC and TDS, with paraben distributions showing compound- and season-specific patterns rather than uniform clusters.

**Figure 5:**
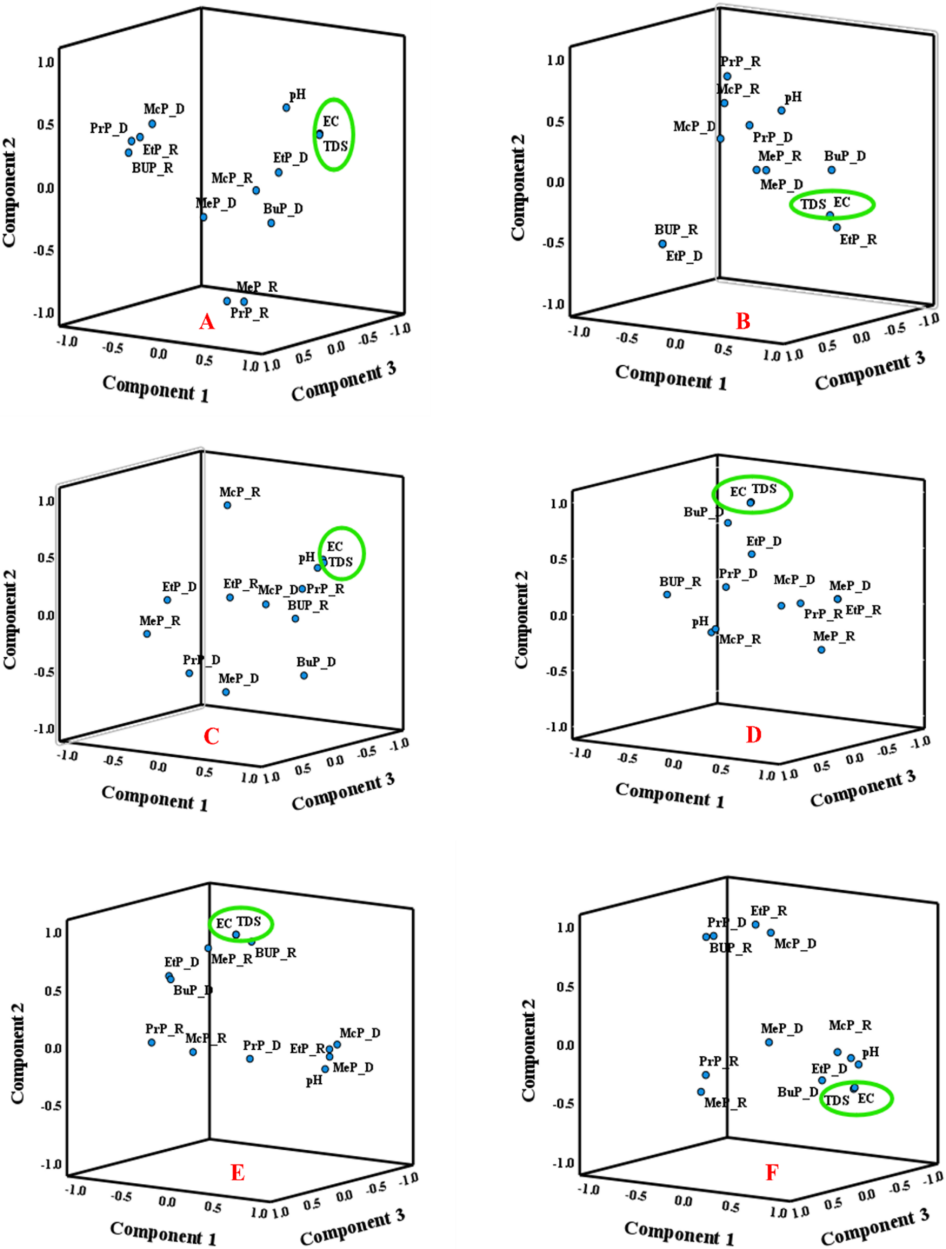
PCA loading 3D plot for Paraben Compounds and studied physicochemical parameters in Osun, Oyo, and Lagos States (A, C, E) rural GW samples (PC1 vs PC2 vs PC3); (B, D, F) urban GW samples (PC1 vs PC2 vs PC3).

#### 3.3.2 Hierarchical Cluster Analysis (HCA)

Hierarchical cluster analysis was applied to assess the similarity patterns among the physicochemical parameters and paraben concentrations in GW samples. Dendrograms were generated with three major consistent clusters (A1, A2, and A3) across the three states, where shorter interpoint distances depicted stronger associations. In Osun GW (Figure 6a­(i,ii)), the dendrogram showed that the paraben variables grouped at relatively low linkage distances, indicating similar occurrence patterns across the data set. The physicochemical parameters formed a separate cluster at higher linkage distances, suggesting weaker similarity to the paraben group. The pH did not join either cluster at the initial stages but merged at an intermediate linkage level, indicating a moderate relationship with both clusters. The final agglomeration step brought all variables together at the longest linkage distance. Similar clustering patterns were observed in the urban Osun data set, indicating consistency in variable relationships across rural and urban sites, as well as highlighting seasonal interactions.^22^ Oyo GW (Figure 6b­(i,ii)) showed similar three-cluster patterns. Parabens formed a cluster at low linkage distances, although some parabens merged earlier than others, indicating variations in their similarity levels. The physicochemical parameters were aggregated into a separate cluster before merging with the paraben cluster at higher linkage distances. The rural and urban dendrograms showed similar cluster structures, suggesting comparable relationships among variables, despite geographic differences. Parabens grouped closely across seasons, indicating co-occurrence and shared pollution source points.

**Figure 6:**
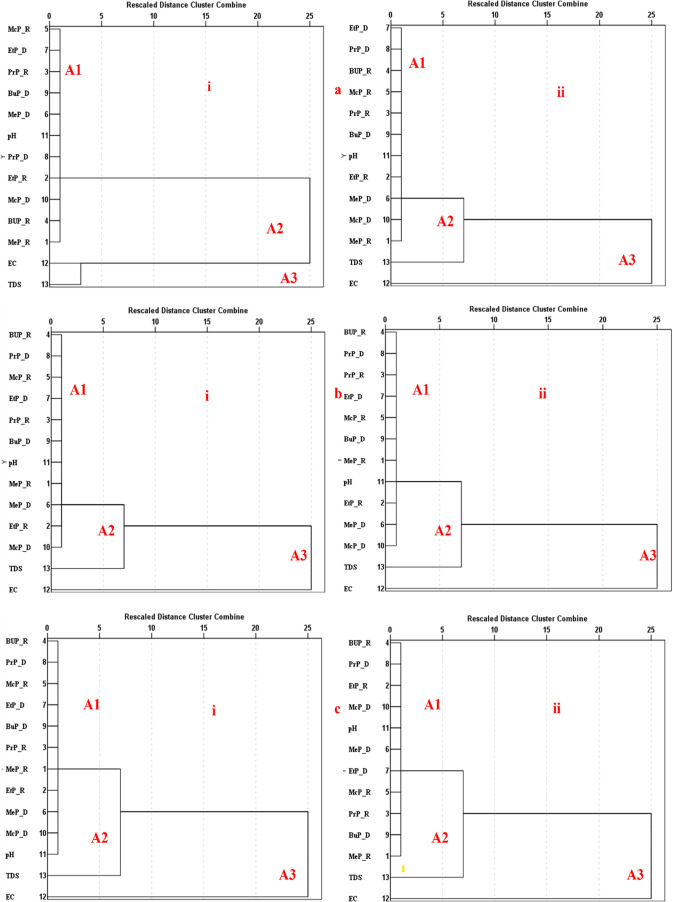
(a–c) Hierarchical cluster analysis (HCA) dendrograms of the relationship between paraben compounds and physicochemical properties in GW from (a), (b), and (c) Lagos during rainy and dry seasons, differentiated by (i) rural and (ii) urban locations.

In Lagos GW (Figure 6c­(i,ii)), rural and urban samples exhibited seasonal clustering patterns similar to those of Oyo. Parabens coclustered across seasons, consistent with global contamination trends. Parabens were grouped at lower linkage distances, while the physicochemical parameters formed a separate cluster that merged later with the paraben cluster. In both rural and urban sites, the order of accumulation differed slightly among paraben types, suggesting some variability in their similarity patterns.^74^ As observed in other states, all variables eventually merged at the highest linkage distance, forming the third cluster. Generally, the HCA results consistently showed two main early forming clusters across states, including a paraben cluster at low linkage distances and a physicochemical parameter cluster at higher linkage distances, with all variables merging at the final stage of agglomeration. These findings confirmed that seasonal variation and physicochemical parameters, particularly EC, TDS, and pH, are key drivers of paraben occurrence and persistence in GW and align with global observations.^39,75,76^

### 3.4 Ecological Risk Assessment

The ecological risk assessment (ERA) of methyl, ethyl, propyl, and butyl parabens was evaluated based on the RQ for the measured mean concentration of GW obtained in this study. Acute and chronic risk exposure of the three taxonomic groups (algae, invertebrates, and fish) to parabens is presented in Table S5. Since there is no PNEC value for McP, it was not computed. The result revealed pronounced spatial and seasonal variation in paraben toxicity across the taxonomic group, with invertebrates consistently exhibiting the highest acute and chronic RQs, particularly during the dry season. This outcome may be attributed to the limited detoxification mechanisms in invertebrates, making them more susceptible to the toxic effects of environmental contaminants. Additionally, the RQ values for paraben compounds exceeded 1 across all sites, with chronic RQs reaching extreme magnitude in Lagos and Osun due to identified source hotspots. This outcome aligns with global observation.^77^

In Osun, acute MeP and BuP HQs exceeded 1 in nearly all samples during the rainy season, with 90–100% of chronic HQs also >1 for algae and invertebrates. PrP generally presented low to moderate risk, with 40–60% of samples within RQ 0.1–1 for algae and fish. Urban GW showed similar patterns, with MeP posing 100% acute and chronic risk across all taxa, while BuP was not detected during the rainy season. However, its absence in urban samples does not negate its potential danger, as previously noted, high concentrations in rural areas demonstrate its persistence and toxicity.

Trends observed in Oyo GW samples indicate that both acute and chronic RQs for all parabens were consistently high in invertebrates and fish. Acute RQ values were >1 for 100% of samples across most concentrations, while chronic RQs for certain taxa, particularly fish, showed more variation, with 40–60% of samples in the 0.1–1.0 range for some compounds. This indicates a persistent eco-toxicological threat across multiple species, especially where agricultural and domestic effluents accumulate with limited dilution in rural aquifers. Compared with its urban counterparts, the RQs revealed a more diverse risk profile. MeP, EtP, PrP, and BuP posed significant acute and chronic risks, especially to algae and invertebrates. Lagos posed the highest ecological risk to all taxa, with over 90% of acute RQs and 70–100% of chronic RQs exceeding 1 for all the paraben variables, reflecting substantial pollutant loading and limited dilution within the GW systems. The potential risks to these organisms were in the order invertebrate > algae > fish, and this corroborates with reported findings within the region.^22^

Across the three states, BuP values were significantly high, and this finding aligns with previously reported studies because of its poor solubility and high lipophilicity.^77,78^ While individual RQs were calculated in this study, a study by Lee et al. suggests that the simultaneous assessment of parabens may reveal even greater ecological risks due to additive or synergistic interactions.^79^ In general, seasonality played a significant role, with dry season amplifying risks across all taxa and regions, likely due to reduced hydrological dilution and increased contaminants load.

### 3.5 Human Exposure and Risk Assessment

Over 80% of the population in the states of Osun, Oyo, and Lagos relies on GW as their drinking and irrigation water supply. Therefore, assessing human exposure to parabens is important when ingesting water from these sources. This study estimated human exposure to parabens *via* estimated daily intake (EDI) and chronic daily intake (CDI) (mg/kg/day) based on the United States Exposure Handbook using measured mean paraben concentrations.^44^ The study focused on children and adults. However, since the established reference dose for McP was unavailable, HQ was not computed; instead, EDI and CDI were evaluated. HQ and CDI values for the studied groups are presented in Table S6, while EDI is illustrated in Figure 7a,b. Also, due to the nonavailability of cancer slope factor (CSF) for paraben compounds, CSF was not evaluated. Generally, EDI values were higher in children than in adults for all paraben compounds in the three states in both the rainy and dry seasons. In this study, adults’ EDI of MeP showed substantial regional variation: in rural Osun State, the EDI peaked at 4.11 mg/kg/day, compared with just 0.11 mg/kg/day in rural Lagos. Despite these differences, all adult values remained comfortably within the 0–10 mg/kg/day Acceptable Daily Intake (ADI) established by the European Food Safety Authority (EFSA) for the combined intake of methyl and ethyl-parabens, as reaffirmed in 2025.^80,81^ In urban settings, Osun still recorded the highest adult EDI, though again it stayed below the ADI threshold. However, exposure among children was markedly higher, particularly in Osun, where rural children reached 9.67 mg/kg/day just below the ADI, and urban children soared to 16.67 mg/kg/day, exceeding the EFSA benchmark. EDI values for EtP, PrP, BuP, and McP were all below 0.8 mg/kg/day for adults and 1.74 mg/kg/day for children. The decreasing order of EDI evaluated in this study, based on their risk profile to humans, fell in the order of MeP > McP > PrP > BuP > EtP, positioning MeP as the most prominent compound affecting both adults and children. Notably, this trend mirrors detection frequencies across the three states highest with Osun, followed by Lagos, and then Oyo, showcasing geographic variance in exposure burden. Worthy of note is that the values reported in this study are of imminent concern because they show an upward trend in paraben exposure *via* water ingestion compared to earlier reports. As expected, the urban areas had higher EDI for all exposure groups, with children being the most affected due to their low body mass, especially in the Osun State, as illustrated in Figure 7a,b.

**Figure 7:**
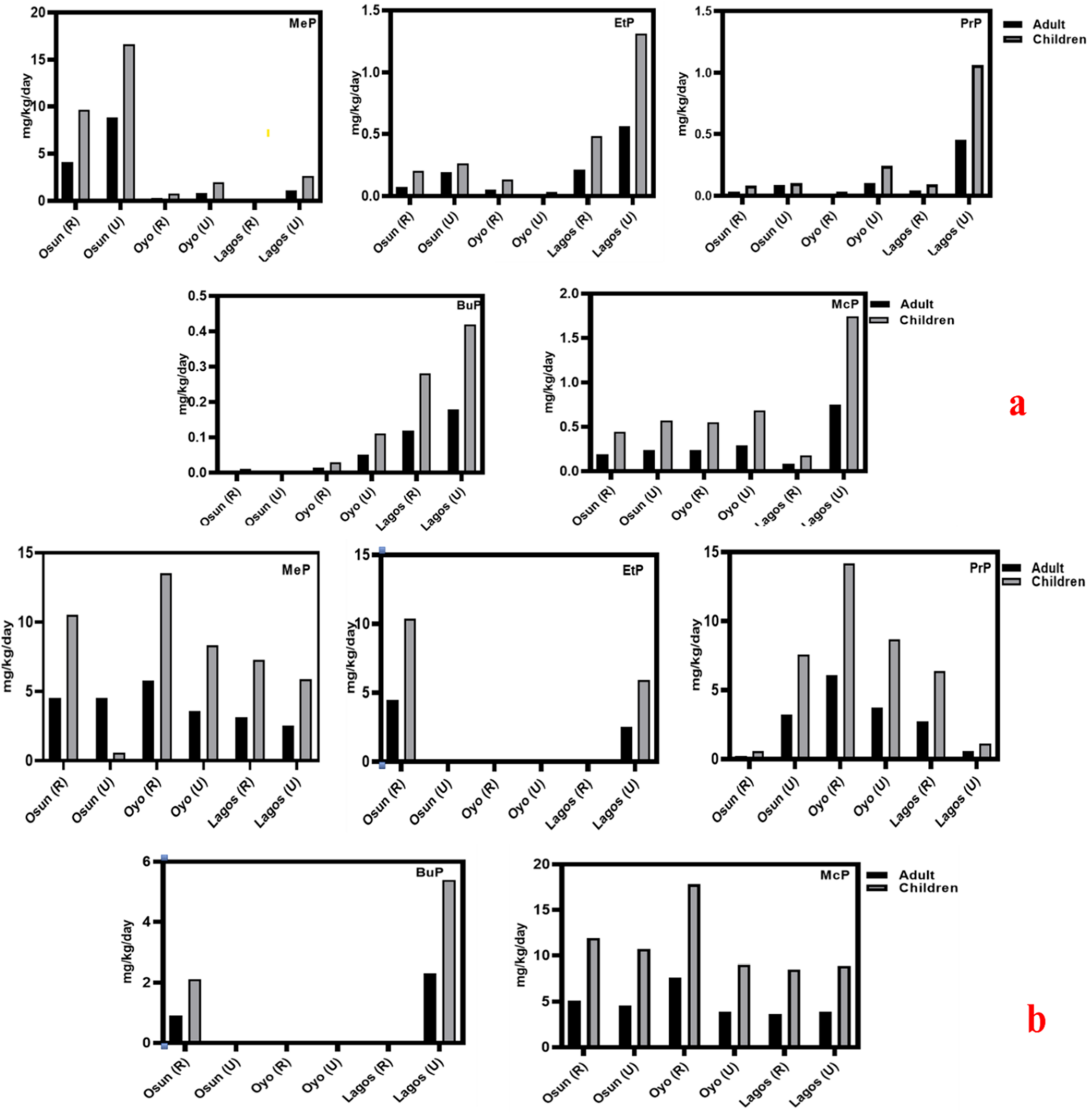
(a, b) Estimated daily intake (EDI) of MeP (methylparaben), EtP (ethylparaben), PrP (propylparaben), BuP (butylparaben), and McP (methyl-3,5-dichloroparaben) in rural (R) and urban (U) areas during the rainy season (a) and dry season (b).

Groundwater serves as the primary source of drinking water for surrounding communities, and the transboundary nature of EDCs necessitates human health risk assessment. To evaluate the noncarcinogenic risks of paraben exposure through water ingestion, hazard quotient (HQ) and chronic daily intake (CDI) were calculated, with the results presented in Table S6. The cumulative hazard index (∑HI) for children and adults was equally calculated in this study and is also presented in Table S6. ∑HI_adult_ for all the paraben compounds ranged from 0.26 to 61.31 across the three states in both rainy and dry seasons, with significantly higher values of ∑HI observed in the urban areas and more pronounced during the dry season. For children, HI values varied significantly across states and seasons, with rainy-season ∑HI_children_ values highest in Lagos urban (12.72) and Osun urban (4.44), indicating high risk. More strikingly, during the dry season, ∑HI_children_ values surged to 143.05 for rural children and 76.69 for urban children, exceeding safe thresholds and signifying severe cumulative exposure from parabens in drinking water. Based on individual parabens, MeP showed moderate to high risk in urban areas and surged during the dry season. EtP was generally below the threshold limit of <1 during the rainy season but exceeded the threshold limit moderately to high during the dry season. Individual HI for PrP in this study was high, and the reason is not far-fetched, as it is the second most commonly used paraben in the region, since its alkyl chain is longer than methyl, the bioaccumulation potential is expected to be higher than MeP. Hence, the higher value and the highest contributor to ∑HI.^82^ While BuP was very negligible to very low during the rainy season, it peaked in the dry season, a pattern that was noticeable for the targeted analytes for all exposure groups. CDI assesses the long-term averaged ingestion exposure and offers a comprehensive insight into potential noncancer health outcomes, such as endocrine disruption and neurological impairment. In this study, CDIs, along with EDI and HQ results, consistently showed higher exposure in children compared with adults, reflecting their lower body weight and greater intake relative to body mass. Importantly, emerging toxicological evidence confirms that chronic exposure to parabens, particularly butylparaben (BuP), can cross the blood–brain barrier, disrupt neuro-behavior and neuro-steroid levels, and impair memory and sensorimotor function in zebrafish models.^83,84^ Furthermore, the elevated CDI in children across the three states not only increases their doses *per* kilograms but also raises serious concerns for their neuroendocrine disruption during this pivotal development phase.

## 4 Conclusion

This study presents the first comprehensive year-long assessment of chlorinated paraben methyl-3,5-dichloro paraben together with methyl, ethyl, propyl, and butyl parabens in groundwater, a major source of drinking water in Nigeria.Methyl-3,5-dichloro paraben (McP) exhibited an almost 100% detection frequency, while methyl paraben (MeP) consistently recorded the highest concentrations across all sampling sites. Osun rural GW samples from the rural areas in the GW region recorded the highest mean paraben concentrations, while Lagos urban areas exhibited the highest concentrations across both rainy and dry seasons. Seasonal dynamics revealed higher concentrations during the dry season, underscoring the role of aquifer recharge and dilution in shaping contaminant persistence. Paraben concentrations exceeded values reported in Nigeria and comparable international studies, reflecting both the local overuse of parabens and a broader global trend of rising antimicrobial-derived contamination. Hierarchical cluster analysis demonstrated that anthropogenic inputs and seasonality strongly govern paraben co-occurrence, while statistical tests revealed no significant difference *(p < 0.05)* between rural and urban sources, indicating that exposure risk is widespread and not restricted to specific geographies. Ecological risk assessment highlighted invertebrates as the most vulnerable taxa, while human health risk analysis revealed children as the most affected demographic, consistent with international evidence of heightened pediatric susceptibility to endocrine disruptors. From these findings, it is evident that a paraben-specific risk assessment framework must be urgently developed and enforced by regulatory bodies to safeguard public health. Given their endocrine-disrupting potential and widespread occurrence, parabens should be prioritized in water treatment and monitoring programs. Importantly, the detection of chlorinated parabens, byproducts of water disinfection known for their greater persistence and enhanced toxicity, highlights the need for further investigation into their environmental fate and health impacts. This knowledge will not only strengthen local water safety policies but also contribute to the global discourse on emerging contaminants, helping to mitigate their growing burden on the overall well-being of the ecosystem.

## Supplementary Material



## Notes

Average pH values of GW in rural and urban areas during the rainy (R) and dry (D) seasons across Osun, Oyo, and Lagos States. Detection frequency of paraben compounds from Lagos, Oyo, and Osun States in Nigeria from rural and urban locations. Physicochemical Characteristics for the Studied Parabens. Coordinates of Sampling Points. Parameters for Evaluation of Ecological Risk Assessment (Chronic and Acute Toxicity) of Paraben in GW. Rotated Component Matrix for Variables in GW Sample from Osun, Oyo, and Lagos States. Seasonal and Geographical Ecological Risk Assessment of Parabens in GW. Human-Health Risk Evaluation to Parabens in GW Samples mg/kg/bw/day. HPLC chromatograms of selected sample sets. Calibration plots of the selected parabens. Raw data sets of selected sampling sites. (PDF)

## ∥

Emmanuel I. Unuabonah was deceased during the preparation of this manuscript.
