# Supplementary material for: Profiling, Distribution, and Risk Assessment of Parabens in Groundwater Near Dumpsites
Source: ACS Omega. 2026 Jan 15;11(3):4085–100. doi: 10.1021/acsomega.5c08260 (PMC12854514; doi:10.1021/acsomega.5c08260)
Supplement: Supplementary file 1 [file ao5c08260_si_001.pdf]

SUPPORTING INFORMATION (SI)

Profiling, distribution, and risk assessment of parabens in groundwater near dumpsites

Esther A. Nnamani[1,2], Oluwaferanmi B. Otitoju[2], Ephraim Akor[1,2], Emmanuel I. Unuabonah[1,2], Martins O. Omorogie[1,2,3]*

[1]*Department of Chemical Sciences, Redeemer's University, PMB 230, PMB 230, Ede, 232101, Nigeria*

[2]*African Centre of Excellence for Water and Environmental Research (ACEWATER), Redeemer's University, PMB 230, Ede, 232101, Nigeria*

[3]*Chair of Urban Water Systems Engineering, Technical University of Munich, Am Coulombwall 3, D-85748, Garching, Germany*

+Emmanuel I. Unuabonah was deceased during the preparation of this manuscript

[*]**Corresponding Author:** omorogiem@run.edu.ng, mo.omorogie@tum.de, dromorogiemoon@gmail.com

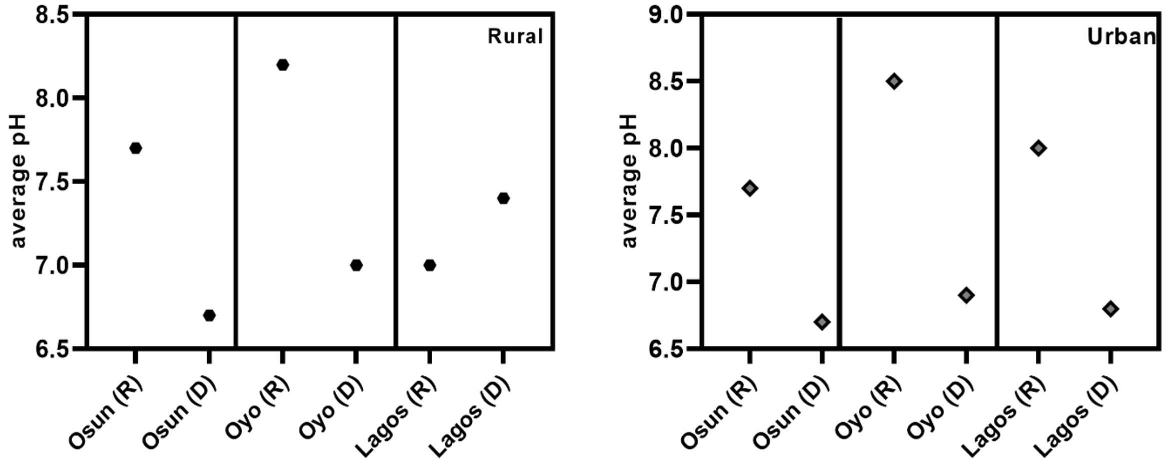

Figure S1: Average pH values of GW in rural and urban areas during the rainy (R) and dry (D) seasons across Osun, Oyo, and Lagos States.

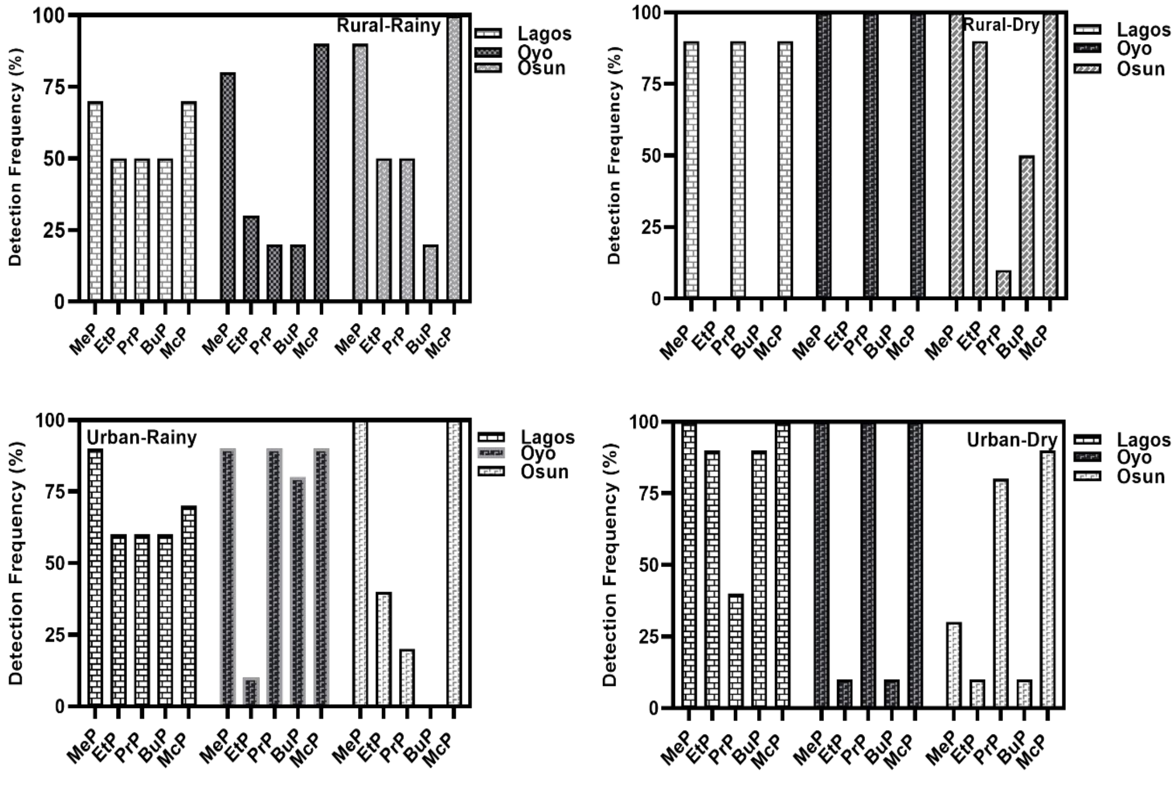

Figure S2: Detection Frequency of Paraben Compounds from Lagos, Oyo, and Osun States in Nigeria from rural and urban locations.

Table S1: Physicochemical Characteristics for the Studied Parabens.

**Paraben derivative**	**Chemical Structure**	**Molecular weight (g/mol)**	**pK_a**	**Water solubility (25°C)**
Methyl paraben (methyl 4-hydroxybenzoate)	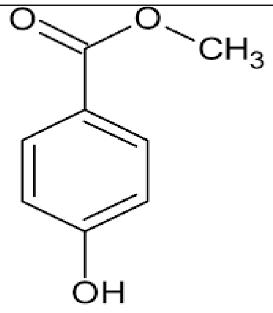	152.15	9.96	2.00 g/100 mL
Ethyl paraben (ethyl 4-hydroxybenzoate)	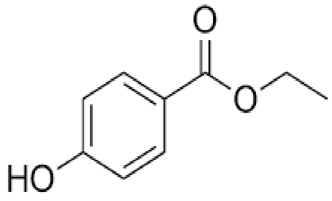	166.17	8.34	0.86 g/100 mL
Propyl paraben (propyl 4-hydroxybenzoate)	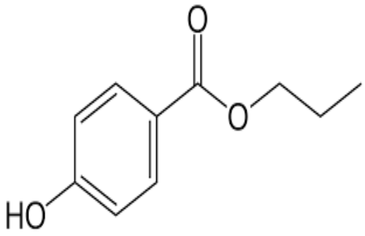	180.21	8.35	0.05 g/100 mL
Butyl paraben (butyl 4-hydroxybenzoate)	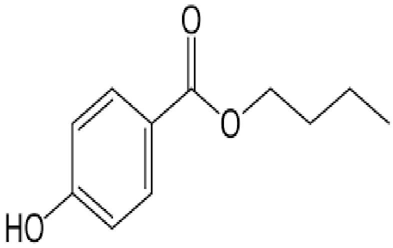	194.23	8.47	0.15 g/100 mL
Methyl 3,5-dichlorobenzoate	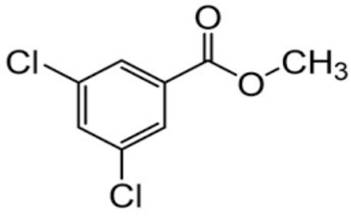	205.03	N/A	0.87-0.90 g/ 100 mL

Table S2: Coordinates of Sampling Points.

State/Setting	Location	Groundwater samples sampled	Coordinates
Osun (Rural)	Ede	Borehole	7°45’37”N, 4°26’29”E
		Borehole	7°44’16”N, 4°25’58”E
		Borehole	7°43 54” N, 4°25’24”E
		Well	7°41’39”N, 4°27’13”E
		Well	7°43’14”N, 4°26’51”E
		Borehole	7°41’51”N, 4°27’18”E
		Well	7°41’57”N, 4°27’32”E
		Well	7°41’47”N, 4°27’10”E
		Borehole	7°41’39”N, 4°27’32”E
		Borehole	7°41’57”N, 4°27’13”E
Osun (Urban)	Osogbo	Well	7°46’14”N, 4°32’17”E
		Borehole	7°46’54”N, 4°32’58”E
		Borehole	7°47’28”N, 4°32’14”E
		Well	7°48’30”N, 4°34’58”E
		Borehole	7°46’28”N, 4°31’59”E
		Borehole	7°46’04”N, 4°32’31”E
		Well	7°47’14”N, 4°32’58”E
		Borehole	7°45’22”N, 4°33’09”E
		Borehole	7°45’26”N, 4°32’59”E
		Borehole	7°41’56”N, 4°32’58”E
Oyo (Rural)	Asejire	Well	7°21’33”N, 4°27’32”E
		Well	7°20’59”N, 4°07’58”E
		Well	7°21’02”N, 4°08’03”E
		Borehole	7°20’59”N, 4°07’58”E
		Borehole	7°21’26”N, 4°08’06”E
		Well	7°21’20”N, 4°08’04”E
		Well	7°21’08”N, 4°08’26”E

		Borehole	7°20’49”N, 4°08’01”E
		Well	7°21’31”N, 4°07’20”E
		Well	7°21’23”N, 4°07’35”E
Oyo (Urban)	Ibadan	Borehole	7°23’22”N, 3°54’32”E
		Borehole	7°23’18”N, 3°54’36”E
		Well	7°23’13”N, 3°54’31”E
		Well	7°23’23”N, 3°54’38”E
		Borehole	7°23’34”N, 3°54’44”E
		Well	7°23’26”N, 3°54’09”E
		Borehole	7°23’17”N, 3°54’20”E
		Borehole	7°22’53”N, 3°53’53”E
		Borehole	7°22’40”N, 3°53’50”E
		Well	7°23’29”N,3 °53’32”E
Lagos (Urban)	Ojota	Borehole	6°35’26” N,3 °22’39” E
		Borehole	6°35’41” N, 3°22’21” E
		Borehole	6°35’25” N, 3°22’25” E
		Borehole	6°35’21” N, 3°22’26” E
		Well	6°35’41” N, 3°22’21” E
		Borehole	6°35’32” N, 3°22’22” E
		Well	6°35’41” N, 3°22’21” E
		Borehole	6°35’30” N, 3°22’28” E
		Well	6°35’15” N, 3°22’25” E
		Borehole	6°35’13” N, 3°22’24” E
Lagos (Rural)	Badagry	Well	6°24’55” N, 2°53’12” E
		Well	6°24’57” N, 2°52’54” E
		Borehole	6°25’18” N, 2°53’54” E
		Well	6°25’23” N, 2°54’44” E
		Borehole	6°26’10” N, 2°54’24” E
		Borehole	6°26’03” N, 2°54’54” E

		Borehole	6°26’06” N, 2°55’59” E
		Well	6°26’08” N, 2°56’34” E
		Borehole	6°26’15” N, 2°53’53” E
		Borehole	6°26’25” N, 2°54’14” E

Table S3: Parameters for Evaluation of Ecological Risk Assessment (Chronic and Acute Toxicity) of Paraben in GW.

Chronic Toxicity

**Analytes**	**Trophic level**	**Toxicity**	**Toxicity (mg/L)**	**PNEC chronic (NOEC/10)**
MeP	Algae	NOEC	12.00	1.20
	Invertebrate	NOEC	0.20	0.02
	Fish	LOEC	25.00	2.50
EtP	Algae	NOEC	2.10	0.21
	Invertebrate	LOEC	2.30	0.23
	Fish	LOEC	17.00	1.70
PrP	Algae	NOEC	2.10	0.21
	Invertebrate	LOEC	0.40	0.04
	Fish	LOEC	2.50	0.25
BuP	Algae	NOEC	0.80	0.08
	Invertebrate	LOEC	0.20	0.02
	Fish	LOEC	1.00	0.10
McP	Fish	LOEC/NOEC	N/A	N/A
	Invertebrate	LOEC/NOEC	N/A	N/A
	Algae	LOEC/NOEC	N/A	N/A

Ref: [1,2]

Acute Toxicity

Analytes	Trophic level	Toxicity	Toxicity (mg/L)	PNEC acute (LC/EC 50)/100
MeP	Algae	LC_{50}	50.00	0.50
	Invertebrate	EC_{50}	11.20	0.11
	Fish	LC_{50}	59.50	0.60
EtP	Algae	LC_{50}	18.00	0.18
	Invertebrate	EC_{50}	20.00	0.20
	Fish	EC_{50}	15.00	0.15
PrP	Algae	LC_{50}	36.00	0.36
	Invertebrate	LC_{50}	15.40	0.15
	Fish	EC_{50}	6.40	0.06
BuP	Algae	LC_{50}	0.06	0.0006
	Invertebrate	LOEC	0.2	0.002
	Fish	EC_{50}	4.20	0.04
McP	Fish	LC_{50}	N/A	N/A
	Invertebrate	LOEC	N/A	N/A
	Algae	EC_{50}	N/A	N/A

Ref: [3]

Table S4: Rotated Component Matrix for Variables in GW Sample from Osun, Oyo, and Lagos States.

	**Osun (R)**			**Osun (U)**			**Oyo (R)**				**Oyo (U)**				**Lagos (R)**			**Lagos (U)**		
	1	2	3	1	2	3	1	2	3	4	1	2	3	4	1	2	3	1	2	3
MeP_R	0.18	-0.94	0.06	-0.33	-0.09	-0.70	-0.45	-0.30	-0.95	-0.5	**0.89**	-0.28	0.02	-0.10	**0.67**	0.45	0.03	0.04	-0.31	**0.93**
EtP_R	-0.65	0.36	**0.51**	**0.80**	-0.36	-0.12	-0.01	0.14	-0.10	**0.89**	**0.95**	0.19	-0.02	0.15	-0.83	0.51	0.13	-0.06	**0.97**	-0.04
PrP_R	0.13	-0.91	0.27	-0.14	**0.83**	0.16	**0.69**	0.35	-0.32	-0.02	**0.53**	0.10	-0.04	-0.73	**0.81**	-0.56	0.01	0.10	-0.16	**0.94**
BuP_R	-0.95	0.17	0.26	-0.49	-0.52	**0.63**	**0.67**	0.12	-0.14	0.16	-0.23	0.24	**0.91**	0.01	0.24	**0.89**	-0.06	-0.58	**0.81**	-0.03
McP_R	**0.70**	0.08	**0.62**	-0.49	-0.52	**0.63**	0.07	**0.87**	0.17	0.19	0.27	-0.12	**0.90**	-0.13	0.09	0.29	-0.35	**0.86**	-0.02	0.04
MeP_D	0.17	-0.17	**0.68**	-0.40	**0.54**	-0.17	-0.31	-0.78	-0.09	-0.20	**0.95**	0.18	-0.02	0.15	-0.86	0.45	0.12	0.14	0.00	0.47
EtP_D	**0.79**	0.21	0.39	-0.49	-0.52	**0.63**	-0.17	0.06	**0.89**	0.07	0.11	**0.52**	0.11	**0.63**	**0.90**	0.16	0.31	**0.96**	-0.07	0.05
PrP_D	-0.93	0.26	0.23	0.12	0.46	0.21	-0.48	-0.64	**0.89**	0.07	-0.38	0.13	-0.21	0.00	-0.03	-0.09	**0.90**	-0.57	**0.81**	-0.13
BuP_D	**0.81**	-0.18	**0.53**	**0.68**	0.09	-0.22	**0.73**	-0.34	-0.01	0.01	-0.18	**0.77**	0.06	0.40	**0.87**	0.15	0.36	**0.84**	-0.23	0.24
McP_D	-0.54	0.47	0.47	0.17	0.42	**0.70**	**0.52**	0.23	0.07	**0.80**	0.37	0.07	0.02	**0.90**	-0.82	0.48	0.18	0.00	**0.88**	-0.18
pH	**0.64**	**0.66**	0.06	0.37	**0.60**	0.10	**0.73**	0.53	-0.63	0.24	0.11	-0.09	**0.72**	0.41	-0.63	-0.21	0.28	**0.53**	-0.27	-0.75
EC	**0.83**	0.43	-0.18	**0.87**	-0.23	0.08	**0.87**	**0.64**	-0.53	0.11	0.04	**0.96**	0.03	-0.05	0.40	**0.90**	-0.01	**0.74**	-0.40	-0.38
TDS	**0.82**	0.43	-0.20	**0.87**	-0.23	0.08	**0.88**	**0.61**	-0.51	0.07	0.03	**0.97**	0.00	-0.04	0.40	**0.89**	-0.10	**0.72**	-0.41	-0.40
Eigen values	6.08	3.06	2.04	3.76	2.54	2.29	4.93	2.20	1.66	1.41	3.45	3.08	2.17	1.74	5.54	3.78	1.28	6.08	3.06	2.04
% of Variance	46.76	23.56	15.7	28.90	19.51	17.59	37.92	16.97	12.76	10.88	26.91	23.70	16.68	13.35	42.62	29.08	9.86	46,76	23.56	15.71
% Cumulative	46.76	70.31	86.02	28.90	48.41	66.00	37.92	54.89	67.65	78.53	26.91	50.61	67.29	80.64	42.61	71.69	81.55	46.76	70.31	86.00

Extraction method: Principal Component Analysis. Rotation method: Varimax Rotation with Kaiser Normalization applied to enhance interpretability. (Factor loadings ≥ 0.5 are highlighted in bold); Figures in bold are those above the acceptable threshold limit of 0.5

Table S5: Seasonal and Geographical Ecological Risk Assessment of Parabens in Groundwater.

Taxonomic group	Paraben	RQ $_{acute}$ range	<0.1 (%)	0.1-1 (%)	1-above (%)	RQ $_{chronic}$ range	<0.1 (%)	0.1-1 (%)	1-above (%)
**Osun Rural (Rainy Season)**									
Algae	MeP	1.35-55.7	0	0	100	0.60-23.2	0	10	90
	EtP	1.40-4.00	0	0	100	1.20-3.50	0	0	100
	PrP	0.10-1.22	0	80	20	0.17-2.08	0	40	60
	BuP	70.09-211.29	0	0	100	0.53-1.58	50	50	0
Invertebrate	MeP	6.03-248.99	0	0	100	33.80-1394.3	0	0	100
	EtP	0.90-4.90	0	10	90	0.15-0.43	0	100	0
	PrP	0.23-2.69	0	40	60	0.88-10.95	0	10	90
	BuP	21.03-63.39	0	0	100	2.10-6.34	0	0	100
Fish	MeP	1.14-46.87	0	0	100	0.30-11.2	0	10	90
	EtP	0.90-4.90	0	90	10	0.15-1.24	0	90	10
	PrP	0.58-7.30	35	65	0	0.14-1.75	60	40	0
	BuP	1.05-3.17	0	0	100	0.42-1.27	0	50	50
**Osun Urban (Rainy Season)**									
Algae	MeP	8.60-74.90	0	0	100	3.60-29.90	0	0	100
	EtP	0.51-1.80	0	20	80	0.44-1.54	0	20	80
	PrP	0.16-0.69	0	100	0	0.28-1.18	0	50	50
	BuP	0.00-0.00	0	0	0	0.00-0.00	0	0	0
Invertebrate	MeP	38.5-320.1	0	0	100	215.7-1792.3	0	0	100
	EtP	0.46-1.62	0	10	90	0.40-1.41	0	10	90
	PrP	0.39-1.65	0	50	50	1.42-6.18	0	0	100
	BuP	0.00-0.00	0	0	0	0.00-0.00	0	0	0
Fish	MeP	7.30-62.90	0	0	100	1.70-15.00	0	0	100
	EtP	0.15-0.54	0	100	0	0.05-0.19	20	80	0
	PrP	0.98-4.12	50	50	0	0.24-0.99	0	100	0
	BuP	0.00-0.00	0	0	0	0.00-0.00	0	0	0
**Oyo Rural (Rainy Season)**									
Algae	MeP	1.95-9.12	0	0	100	0.81-3.80	0	10	90
	EtP	2.39-4.30	0	0	100	2.05-3.68	0	0	100
	PrP	1.57-1.75	0	0	100	5.90-6.57	0	0	100

	BuP	20.11-230.69	0	0	100	0.50-5.76	0	0	100
Invertebrate	MeP	11.67-40.69	0	0	100	48.79-227.88	0	0	100
	EtP	2.15-3.86	0	0	100	0.25-0.45	0	100	0
	PrP	3.93-4.38	0	0	100	0.94-1.05	0	50	50
	BuP	20.11-230.69	0	0	100	2.01-23.06	0	0	100
Fish	MeP	2.87-7.66	0	0	100	0.39-1.82	0	60	40
	EtP	2.87-5.15	0	0	100	0.25-0.45	0	100	0
	PrP	3.93-4.38	0	0	100	0.9-1.05	0	50	50
	BuP	0.46-1.01	0	50	50	0.40-4.61	0	50	50
**Oyo Urban (Rainy Season)**									
Algae	MeP	1.70-16.88	0	0	100	0.71-7.03	0	10	90
	EtP	0.00-2.12	0	0	100	0.00-1.82	0	0	100
	PrP	0.26-3.31	0	50	50	0.44-5.67	0	60	40
	BuP	17.67-691.82	0	0	100	0.13-5.19	0	10	90
Invertebrate	MeP	7.58-75.34	0	0	100	42.43-421.93	0	0	100
	EtP	0.00-1.91	0	0	100	0.00-7.89	0	0	100
	PrP	0.55-7.93	0	40	60	2.32-29.76	0	0	100
	BuP	5.30-166.12	0	0	100	0.53-20.75	0	10	90
Fish	MeP	1.43-8.53	0	0	100	0.34-3.38	0	60	40
	EtP	0.00-2.54	0	0	100	0.00-4.64	0	0	100
	PrP	1.55-19.84	0	0	100	0.33-4.76	0	60	40
	BuP	0.26-10.37	0	10	90	0.11-3.80	0	40	60
**Lagos Rural (Rainy Season)**									
Algae	MeP	0.00-7.83	0	0	100	0.64-2.67	30	70	0
	EtP	4.37-19.99	0	0	100	3.75-17.14	0	0	100
	PrP	0.48-3.43	0	40	60	0.84-5.88	0	70	30
	BuP	718.09-1181.87	0	0	100	5.38-13.96	0	0	100
Invertebrate	MeP	0.00-28.57	0	10	90	46.62-195.65	0	0	100
	EtP	3.93-17.99	0	0	100	3.42-15.65	0	0	100
	PrP	1.16-8.23	0	0	100	4.38-30.89	0	0	100
	BuP	215.43-558.56	0	0	100	21.54-55.85	0	0	100

Fish	MeP	0.00-6.58	0	0	100	0.00-1.56	0	60	40
	EtP	5.25-23.99	0	0	100	0.46-2.11	0	50	50
	PrP	2.92-20.59	0	0	100	0.70-4.94	0	20	80
	BuP	10.77-26.93	0	0	100	4.31-11.17	0	0	100
**Lagos Urban (Rainy Season)**									
Algae	MeP	2.89-11.60	0	0	100	1.21-18.14	0	0	100
	EtP	8.04-32.23	0	0	100	6.88-27.63	0	0	100
	PrP	0.90-14.91	0	10	90	1.54-25.55	0	0	100
	BuP	716.21-6627.37	0	0	100	5.37-49.71	0	0	100
Invertebrate	MeP	2.89-11.60	0	0	100	72.34-1088.36	0	0	100
	EtP	7.23-29.02	0	0	100	6.29-25.23	0	0	100
	PrP	2.16-35.77	0	0	100	8.11-134.15	0	0	100
	BuP	3214.86-1988.21	0	0	100	21.48-198.82	0	0	100
Fish	MeP	2.43-36.58	0	0	100	0.57-8.71	0	30	70
	EtP	9.64-38.68	0	0	100	0.85-3.41	0	10	90
	PrP	23.38-65.03	0	0	100	1.29-21.46	0	0	100
	BuP	10.74-99.41	0	0	100	6.81-39.76	0	0	100
**Osun Rural (Dry Season)**									
Algae	MeP	11.99-40.36	0	0	100	4.99-16.82	0	0	100
	EtP	30.92-122.56	0	0	100	26.50-105.05	0	0	100
	PrP	0.00-22.91	0	0	100	0.00-39.27	0	0	100
	BuP	67.42-151.77	0	0	100	64.68-113.83	0	0	100
Invertebrate	MeP	53.55-180.18	0	0	100	299.88-1009.04	0	0	100
	EtP	27.82-110.30	0	0	100	24.19-95.91	0	0	100
	PrP	0.00-55.00	0	0	100	0.00-206.25	0	0	100
	BuP	20.02-45.53	0	0	100	202.26-455.33	0	0	100
Fish	MeP	10.08-33.91	0	0	100	2.39-8.07	0	0	100
	EtP	37.09-	0	0	100	3.27-12.97	0	0	100

		131.91							
	PrP	0.00-137.50	0	0	100	0.00-33.00	0	0	100
	BuP	101.13-169.95	0	0	100	40.45-91.06	0	0	100
**Osun Urban (Dry Season)**									
Algae	MeP	22.35-55.82	0	0	100	9.32-23.26	0	0	100
	EtP	0.00-0.00	0	0	0	0.00-0.00	0	0	0
	PrP	22.11-64.94	0	0	100	37.92-111.33	0	0	100
	BuP	0.00-0.00	0	0	0	0.00-0.00	0	0	0
Invertebrate	MeP	99.81-249.21	0	0	100	558.92-1395.59	0	0	100
	EtP	0.00-0.00	0	0	0	0.00-0.00	0	0	0
	PrP	53.08-155.86	0	0	100	199.07-584.48	0	0	100
	BuP	0.00-0.00	0	0	0	0.00-0.00	0	0	0
Fish	MeP	18.78-39.51	0	0	100	4.47-11.16	0	0	100
	EtP	0.00-0.00	0	0	0	0.00-0.00	0	0	0
	PrP	132.72-389.66	0	0	100	31.85-79.45	0	0	100
	BuP	0.00-0.00	0	0	0	0.00-0.00	0	0	0
**Oyo Rural (Dry Season)**									
Algae	MeP	36.57-46.23	0	0	100	12.83-19.53	0	0	100
	EtP	0.00-0.00	0	0	0	0.00-0.00	0	0	0
	PrP	44.76-67.57	0	0	100	76.73-115.84	0	0	100
	BuP	0.00-0.00	0	0	0	0.00-0.00	0	0	0
Invertebrate	MeP	137.45-209.25	0	0	100	769.75-1120.70	0	0	100
	EtP	0.00-0.00	0	0	0	0.00-0.00	0	0	0
	PrP	120.12-162.17	0	0	100	402.81-602.63	0	0	100
	BuP	0.00-0.00	0	0	0	0.00-0.00	0	0	0
Fish	MeP	25.87-39.98	0	0	100	6.15-9.37	0	0	100
	EtP	0.00-0.00	0	0	0	0.00-0.00	0	0	0
	PrP	300.31-405.43	0	0	100	64.45-97.30	0	0	100
	BuP	0.00-0.00	0	0	0	0.00-0.00	0	0	0

**Oyo Urban (Dry Season)**									
Algae	MeP	0.97-44.86	0	10	90	0.40-16.97	0	10	90
	EtP	0.00-0.00	0	0	0	0.00-0.00	0	0	0
	PrP	1.77-64.79	0	0	100	3.03-111.07	0	0	100
	BuP	0.00-0.00	0	0	0	0.00-0.00	0	0	0
Invertebrate	MeP	4.33-200.29	0	0	100	24.28-1121.60	0	0	100
	EtP	0.00-0.00	0	0	0	0.00-0.00	0	0	0
	PrP	4.24-141.04	0	0	100	15.91-583.15	0	0	100
	BuP	0.00-0.00	0	0	0	0.00-0.00	0	0	0
Fish	MeP	0.82-37.70	0	10	90	0.19-8.97	0	10	90
	EtP	0.00-0.00	0	0	0	0.00-0.00	0	0	0
	PrP	10.60-388.76	0	0	100	2.54-93.30	0	0	100
	BuP	0.00-0.00	0	0	0	0.00-0.00	0	0	0
**Lagos Rural (Dry Season)**									
Algae	MeP	5.29-27.45	0	0	100	4.25-11.44	0	0	100
	EtP	17.70-82.35	0	0	100	15.17-70.58	0	0	100
	PrP	2.00-3.86	0	0	100	3.44-6.62	0	0	100
	BuP	774.25-2406.98	0	0	100	58.08-180.48	0	0	100
Invertebrate	MeP	23.63-122.56	0	0	100	132.34-686.33	0	0	100
	EtP	15.93-74.11	0	0	100	13.85-64.44	0	0	100
	PrP	4.81-9.27	0	0	100	18.04-34.75	0	0	100
	BuP	2563.36-6340.95	0	0	100	172.41-721.92	0	0	100
Fish	MeP	4.45-23.07	0	0	100	1.06-5.49	0	0	100
	EtP	21.24-98.82	0	0	100	1.87-8.72	0	0	100
	PrP	12.03-23.16	0	0	100	2.89-5.56	0	0	100
	BuP	86.20-360.96	0	0	100	51.27-144.38	0	0	100
**Lagos Urban (Dry Season)**									
Algae	MeP	12.60-28.26	0	0	100	5.25-13.24	0	0	100
	EtP	0.00-0.00	0	0	0	0.00-0.00	0	0	0
	PrP	14.95-38.85	0	0	100	25.62-66.60	0	0	100

	BuP	0.00-0.00	0	0	0	0.00-0.00	0	0	0
Invertebrate	MeP	56.23-141.87	0	0	100	314.91-794.49	0	0	100
	EtP	0.00-0.00	0	0	0	0.00-0.00	0	0	0
	PrP	35.87-93.24	0	0	100	134.52-349.64	0	0	100
	BuP	0.00-0.00	0	0	0	0.00-0.00	0	0	0
Fish	MeP	10.58-23.74	0	0	100	2.52-6.36	0	0	100
	EtP	0.00-0.00	0	0	0	0.00-0.00	0	0	0
	PrP	89.68-233.09	0	0	100	21.52-55.94	0	0	100
	BuP	0.00-0.00	0	0	0	0.00-0.00	0	0	0

Table S6: Human-Health Risk Evaluation to Parabens in GW Samples mg/kg/bw/day.

Location	Season	Analyte	Human	CDI	HQ	LoR	$\sum HI_{adult}$	$\sum HI_{children}$
Osun (Rural)	Rainy	MeP	Adult	8.23	0.42	Low	1.17	1.81
			Children	9.61	0.96	Mid		
		EtP	Adult	0.0004	0.007	Low		
			Children	0.17	0.017	Low		
		PrP	Adult	0.06	0.33	Low		
			Children	0.07	0.76	Low		
		BuP	Adult	0.009	0.03	Low		
			Children	0.011	0.07	Low		
		McP	Adult	0.38	**	**		
			Children	0.44	**	**		
Osun (Urban)	Rainy	MeP	Adult	24.04	1.68	High	2.55	4.44
			Children	40.88	3.36	High		
		EtP	Adult	0.22	0.02	Low		
			Children	0.28	0.03	Low		
		PrP	Adult	0.09	0.85	Mid		
			Children	0.11	1.05	High		
		BuP	Adult	*	*	*		
			Children	*	*	*		
		McP	Adult	0.49	**	**		
			Children	0.57	**	**		
Oyo (Rural)	Rainy	MeP	Adult	0.65	0.03	Low	0.26	0.63
			Children	0.65	0.08	Low		
		EtP	Adult	0.11	0.005	Low		
			Children	0.13	0.01	Low		
		PrP	Adult	0.03	0.14	Low		
			Children	0.07	0.33	Low		
		BuP	Adult	0.02	0.08	Low		
			Children	0.03	0.21	Low		
		McP	Adult	0.47	**	**		
			Children	0.55	**	**		
Oyo (Urban)	Rainy	MeP	Adult	1.67	0.08	Low	1.40	5.65
			Children	1.94	0.19	Low		
		EtP	Adult	0.007	0.001	Low		
			Children	0.025	0.003	Low		

		PrP	Adult	0.20	1.01	High		
			Children	0.73	4.74	High		
		Bup	Adult	0.10	0.31	Low		
			Children	0.12	0.72	Low		
		McP	Adult	0.53	**	**		
			Children	0.68	**	**		
Lagos (Rural)	Rainy	MeP	Adult	0.16	0.01	Low	1.19	2.77
			Children	0.18	0.02	Low		
		EtP	Adult	0.41	0.02	Low		
			Children	0.48	0.05	Low		
		PrP	Adult	0.14	0.40	Low		
			Children	0.160	0.94	Mid		
		BuP	Adult	0.24	0.76	Low		
			Children	0.28	1.76	High		
		McP	Adult	0.15	**	**		
			Children	0.18	**	**		
Lagos(Urban)	Rainy	MeP	Adult	2.22	0.11	Low	5.83	12.72
			Children	2.60	0.26	Low		
		EtP	Adult	1.12	0.06	Low		
			Children	1.31	0.13	Low		
		PrP	Adult	0.91	4.53	High		
			Children	1.06	10.58	High		
		BuP	Adult	0.36	1.13	High		
			Children	0.42	1.75	High		
		McP	Adult	1.49	**	**		
			Children	1.74	**	**		
Osun (Rural)	Dry	MeP	Adult	9.01	0.45	Low	12.89	20.73
			Children	10.52	1.05	High		
		EtP	Adult	8.89	4.45	High		
			Children	10.38	1.04	High		
		PrP	Adult	0.47	2.36	High		
			Children	0.55	5.50	High		
		BuP	Adult	1.80	5.63	High		
			Children	2.10	13.14	High		
		McP	Adult	10.23	**	**		
			Children	11.93	**	**		

Osun (Urban)	Dry	MeP	Adult	9.08	0.45	Low	32.86	76.69
			Children	10.60	1.06	High		
		EtP	Adult	*	*	*		
			Children	*	*	*		
		PrP	Adult	6.48	32.41	High		
			Children	7.56	75.63	High		
		BuP	Adult	*	*	*		
			Children	*	*	*		
		McP	Adult	9.18	**	**		
			Children	10.71	**	**		
Oyo (Rural)	Dry	MeP	Adult	11.58	0.58	Low	61.31	143.05
			Children	13.51	1.35	High		
		EtP	Adult	*	*	*		
			Children	*	*	*		
		PrP	Adult	12.14	60.73	High		
			Children	14.17	141.70	High		
		BuP	Adult	*	*	*		
			Children	*	*	*		
		McP	Adult	15.27	**	**		
			Children	17.82	**	**		
Oyo (Urban)	Dry	MeP	Adult	7.15	0.36	Low	37.62	87.78
			Children	8.34	0.84	Low		
		EtP	Adult	*	*	*		
			Children	*	*	*		
		PrP	Adult	7.45	37.26	High		
			Children	8.69	86.94	High		
		BuP	Adult	*	*	*		
			Children	*	*	*		
		McP	Adult	7.77	**	**		
			Children	9.06	**	**		
Lagos (Rural)	Dry	MeP	Adult	6.24	0.32	Low	27.57	64.32
			Children	7.27	0.73	Low		
		EtP	Adult	*	*	*		
			Children	*	*	*		
		PrP	Adult	5.45	27.25	High		
			Children	6.36	63.59	High		

		BuP	Adult	*	*	*		
			Children	*	*	*		
		McP	Adult	7.30	**	**		
			Children	8.51	**	**		
Lagos (Urban)	Dry	MeP	Adult	5.03	0.25	Low	15.52	36.36
			Children	5.86	0.73	Low		
		EtP	Adult	5.11	0.26	Low		
			Children	5.94	0.60	Low		
		PrP	Adult	0.12	0.60	Low		
			Children	0.14	1.41	High		
		BuP	Adult	4.62	14.41	High		
			Children	5.38	33.62	High		
		McP	Adult	5.66	**	**		
			Children	6.60	**	**		

CDI (Chronic daily intake); HQ (Hazard quotient); HI (Hazard index); *(No value); ** (No RfD value for McP); LoR (Level of risk).

)ata File C:\CHEM32\1\DATA\ESTHER PARABENS (5) 2025-02-17 21-19-28\OS1.D
Sample Name: AS1

===

Acq. Operator	: Akor Ephriam	Seq. Line :	1
Acq. Instrument	: Instrument 1	Location :	P1-A-02
Injection Date	: 2/17/2025 9:20:14 PM	Inj :	1
		Inj Volume :	20.000 µl

Acq. Method : C:\CHEM32\1\DATA\ESTHER PARABENS (5) 2025-02-17 21-19-28\ESTHER PARABENS C2.M
Last changed : 2/17/2025 9:19:28 PM by Akor Ephriam
Analysis Method : C:\CHEM32\1\METHODS\ESTHER PARABENS C2.M
Last changed : 2/17/2025 9:19:28 PM by Akor Ephriam

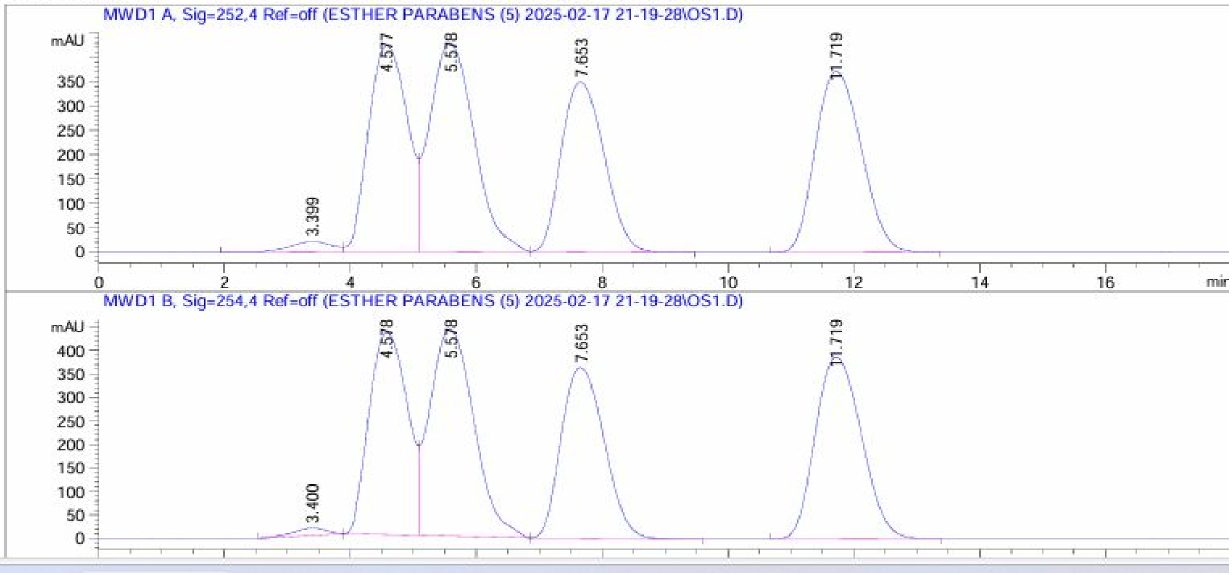

Figure S3: Chromatograms showing the retention time for five Paraben Compounds.

```
Data File C:\CHEM32\1\DATA\ESTHER PARABENS (5) 2025-02-17 21-19-28\EDE5.D
Sample Name: IB1

    =====================================================================
    Acq. Operator   : Akor Ephriam                    Seq. Line :  11
    Acq. Instrument : Instrument 1                     Location : P1-B-03
    Injection Date  : 2/18/2025 12:29:27 AM                 Inj :   1
                                                     Inj Volume : 20.000 µl
    Acq. Method     : C:\CHEM32\1\DATA\ESTHER PARABENS (5) 2025-02-17 21-19-28\ESTHER PARABENS
                      C2.M
    Last changed    : 2/17/2025 9:19:28 PM by Akor Ephriam
    Analysis Method : C:\CHEM32\1\METHODS\ESTHER PARABENS C2.M
    Last changed    : 2/17/2025 9:19:28 PM by Akor Ephriam
```

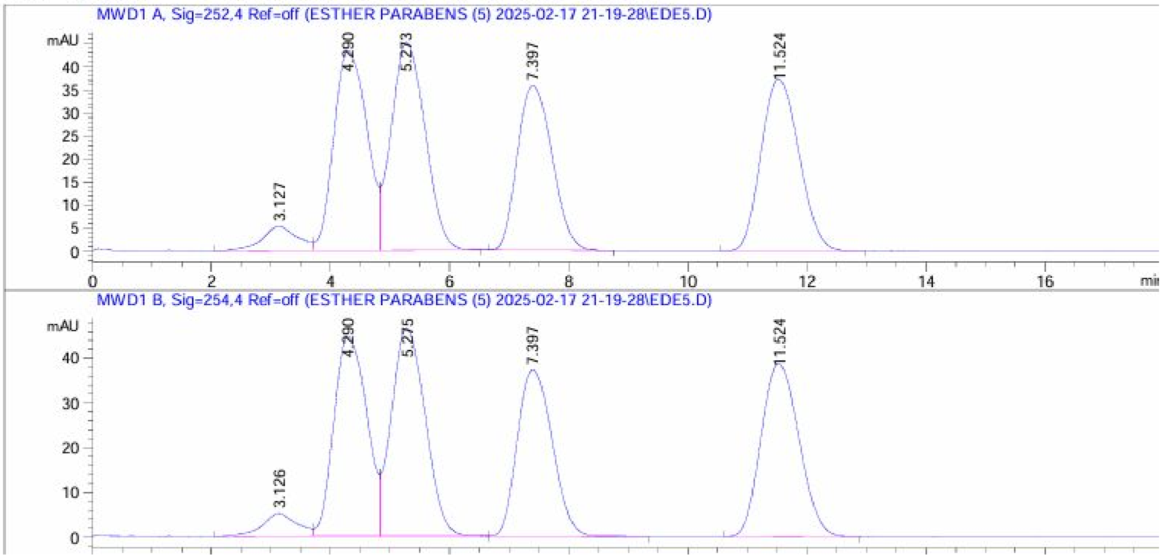

Figure S4: Chromatograms showing the retention time for five Paraben Compounds.

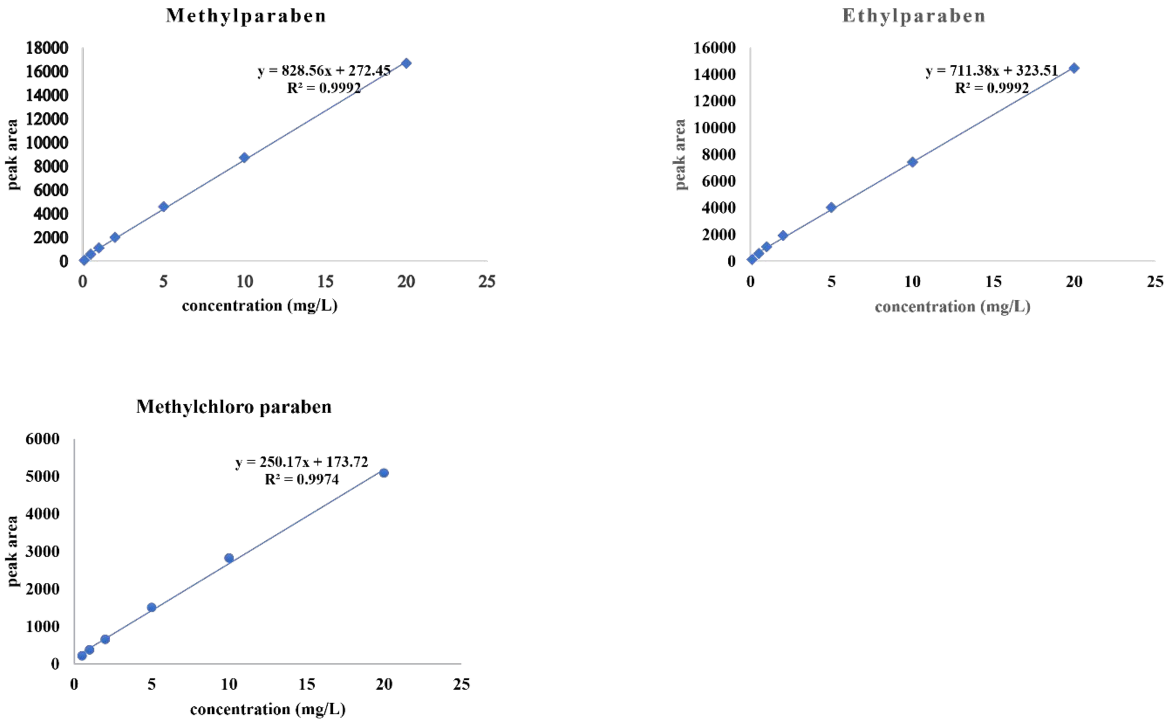

Figure S5: Calibration curves for Methylparaben, Ethylparaben and Methylchloro paraben.

Table S7: Osun State rural samples for Methylparaben, Ethylparaben, Propylparaben, Butylparaben and Methylchloro paraben during rain and dry seasons.

	OSUN RURAL SAMPLES									
SAMPLE ID	MeP (R)	MeP (D)	EtP (R)	EtP (D)	PrP (R)	PrP (D)	BuP (R)	BuP (D)	McP (R)	McP (D)
A	-	17.66	-	19.75	-	-	-	16.80	1.36	16.32
B	26.49	10.70	-	-	-	8.25	-	-	0.54	12.69
C	20.00	6.00	-	5.56	0.04	-	-	5.17	0.48	6.81
D	0.68	15.24	-	16.16	-	-	-	14.05	0.46	14.83
E	10.57	16.25	0.52	16.55	0.40	-	-	16.41	0.69	18.37
F	26.43	19.87	-	19.74	-	-	-	19.11	0.90	22.16
G	27.89	17.88	0.55	19.05	-	-	-	-	1.13	21.90
H	7.42	20.18	0.26	12.06	0.23	-	-	-	0.06	22.06
I	15.00	15.64	0.73	17.08	0.04	-	0.13	-	0.28	20.22
J	19.68	18.33	0.44	19.79	0.44	-	0.04	-	0.69	23.60

Table S8: Oyo State rural samples for Methylparaben, Ethylparaben, Propylparaben, Butylparaben and Methylchloro paraben during rain and dry seasons.

	OYO URBAN SAMPLES									
SAMPLE ID	MeP (R)	MeP (D)	EtP (R)	EtP (D)	PrP (R)	PrP (D)	BuP (R)	BuP (D)	McP (R)	McP (D)
A	0.85	0.49	-	-	0.10	0.64	-	-	0.06	1.06
B	1.32	9.41	-	-	1.19	9.91	0.33	-	1.52	13.30
C	1.52	8.68	-	-	0.08	9.07	0.12	-	1.36	12.55
D	5.84	19.28	-	-	0.37	20.02	0.30	-	0.51	26.80
E	2.19	20.37	-	-	0.17	21.16	0.38	-	2.02	28.46
F	1.63	11.32	-	-	0.09	11.80	0.42	-	1.42	15.32
G	2.29	7.19	-	-	0.18	7.55	0.08	-	2.08	10.79
H	5.08	22.43	0.38	-	0.49	23.33	0.08	-	0.72	0.58
I	8.44	15.20	-	-	0.88	15.83	0.01	-	0.50	20.26
J	-	10.68	-	-	-	11.11	-	-	-	6.86

References

(1) Yamamoto, H.; Tamura, I.; Hirata, Y.; Kato, J.; Kagota, K.; Katsuki, S.; Yamamoto, A.; Kagami, Y.; Tatarazako, N. Aquatic toxicity and ecological risk assessment of seven parabens: individual and additive approach. *Science of the Total Environment* **2011**, *410*, 102-111.

(2) Bolujoko, N. B.; Ogunlaja, O. O.; Alfred, M. O.; Okewole, D. M.; Ogunlaja, A.; Olukanni, O. D.; Msagati, T. A.; Unuabonah, E. I. Occurrence and human exposure assessment of parabens in water sources in Osun State, Nigeria. *Science of The Total Environment* **2022**, *814*, 152448.

(3) Fu, L.; Sun, Y.; Zhou, J.; Li, H.; Liang, S.-x. Parabens, triclosan and bisphenol A in surface waters and sediments of baiyang lake, China: occurrence, distribution, and potential risk assessment. *Toxics* **2023**, *12*, 31.
